# miR-137–LAPTM4B regulates cytoskeleton organization and cancer metastasis via the RhoA-LIMK-Cofilin pathway in osteosarcoma

**DOI:** 10.1038/s41389-023-00471-5

**Published:** 2023-05-06

**Authors:** Ruyu Yan, Dan Liu, Junjie Wang, Minxia Liu, Hongjuan Guo, Jing Bai, Shuo Yang, Jun Chang, Zhihong Yao, Zuozhang Yang, Tomas Blom, Kecheng Zhou

**Affiliations:** 1 School of Life Sciences, Anhui Medical University, Hefei, 230032 China; 2 Institute for Molecular Medicine Finland, Helsinki Institute of Life Science, University of Helsinki, Helsinki, 00290 Finland; 3 Department of Orthopaedics, The First Affiliated Hospital of Anhui Medical University, Hefei, 230032 China; 4 Bone and Soft Tissue Tumours Research Centre of Yunnan Province, Department of Orthopaedics, The Third Affiliated Hospital of Kunming Medical University (Yunnan Cancer Hospital, Yunnan Cancer Center), Kunming, Yunnan 650118 China; 5 Department of Anatomy, Faculty of Medicine, University of Helsinki, Helsinki, 00014 Finland; 6 Minerva Foundation Institute for Medical Research, Helsinki, 00014 Finland

**Keywords:** Cytoskeleton, Metastasis

## Abstract

Osteosarcoma (OS) is a rare malignant bone tumor but is one leading cause of cancer mortality in childhood and adolescence. Cancer metastasis accounts for the primary reason for treatment failure in OS patients. The dynamic organization of the cytoskeleton is fundamental for cell motility, migration, and cancer metastasis. Lysosome Associated Protein Transmembrane 4B (LAPTM4B) is an oncogene participating in various biological progress central to cancer biogenesis. However, the potential roles of LAPTM4B in OS and the related mechanisms remain unknown. Here, we established the elevated LAPTM4B expression in OS, and it is essential in regulating stress fiber organization through RhoA–LIMK–cofilin signaling pathway. In terms of mechanism, our data revealed that LAPTM4B promotes RhoA protein stability by suppressing the ubiquitin-mediated proteasome degradation pathway. Moreover, our data show that miR-137, rather than gene copy number and methylation status, contributes to the upregulation of LAPTM4B in OS. We report that miR-137 is capable of regulating stress fiber arrangement, OS cell migration, and metastasis via targeting LAPTM4B. Combining results from cells, patients’ tissue samples, the animal model, and cancer databases, this study further suggests that the miR-137–LAPTM4B axis represents a clinically relevant pathway in OS progression and a viable target for novel therapeutics.

## Introduction

Osteosarcoma (OS) is a rare malignant bone tumor derived from osteoblasts but represents the leading cause of cancer mortality in childhood and adolescence, with a 5-year survival rate of ~20% for patients with metastatic or relapsed OS [1]. Novel techniques, *e.g*. X-ray and Magnetic Resonance Imaging (MRI), have clearly benefited the disease diagnosis. However, few potential targets have been identified in OS, and there have rarely been major improvements in clinical treatments for OS over the past decades [1], neoadjuvant chemotherapeutics combined with surgery is still the most commonly employed treatment. Cancer metastasis is the primary cause of treatment failure in OS patients [2], thus deciphering the underpinning molecular mechanisms may develop novel targeted therapeutics for OS.

Cell motility, migration, and cancer metastasis depend on a dynamic cytoskeleton that is regulated by the crosstalk among multiple signaling pathways [3]. Stress fibers are cytoskeletal structures providing force and support for cell migration and adhesion, the organization of which was reported promoting cell stiffening and proliferation in cancer cells [4]. Stress fibers are contractile structures composed of actin, myosin, as well as crosslinking proteins, and undergoes dynamic assembly and disassembly [5]. Several instrumental molecules in the regulation of cytoskeleton arrangement have been identified, with small GTPases Rac1, Cdc42, and RhoA in the central position [6–8]. Rac1 stimulates lamellipodia and membrane ruffle formation via activating the actin nucleating complex, whereas Cdc42 induces filopodia formation, pointing to the direction of cell migration [5]. RhoA directly promotes stress fiber formation via the LIM kinase (LIMK)-cofilin pathway. Specifically, cofilin acts as an actin-binding protein, essential for actin filament depolymerization [8, 9], and LIMK adjusts actin dynamics via regulating the phosphorylation of cofilin. By promoting LIMK phosphorylation, RhoA abolishes the actin-binding activity of cofilin and further enhances the formation of stress fiber [9].

Lysosome Associated Protein Transmembrane 4B (LAPTM4B) was originally cloned from hepatocellular carcinoma [10]. Elevated LAPTM4B expression was observed in acute leukemia [11] and several other solid cancers [12, 13], and functionally has been involved in regulating stemness [11], drug resistance [12, 14], autophagy [13], as well as migration and metastasis [15]. On the molecular level, LAPTM4B interacts and stabilizes the Epidermal Growth Factor Receptor (EGFR) at endosomes, suppresses EGF induced EGFR sorting and lysosomal degradation, prolonging the EGFR signaling [16, 17]. LAPTM4B has been shown to regulate the subcellular ceramide pool [18–20] and to interact with the leucine transporter CD98hc [21, 22], regulating cellular nutrient signaling [21–23]. Recently, we identified that LAPTM4B promotes integrin beta1 recycling and focal adhesion dynamics, critical progress promoting skin cancer development [24]. However, LAPTM4B has not been well investigated in OS hitherto, albeit two pilot studies suggest the high expression of LAPTM4B in OS [25, 26]. The detailed mechanism underpinning LAPTM4B dysregulation in OS is not recognized, besides, the functional phenotype and relevant mechanisms remain unknown.

Genome instability/altered gene amplification serve as one of novel cancer hallmarks [27], and gene copy number variations (CNV) and methylation status represent major reasons driving abnormal gene expression [28, 29]. Alternatively, post-transcriptional regulation, such as MicroRNAs (miRNAs), provides another mechanism of dysregulation. For instance, miR-155, miR-944, and miR-129* participate in lung cancer cell proliferation, tumor growth [30, 31], cancer metastasis [32], respectively. The interplay between miRNAs and oncogenes or tumor suppressor genes has been proposed as a complex network, which is prevalent and functional in cancers [33].

In the current study, we deep mined publicly available databases and examined OS cells and tissues, to gain insight into the regulation and function of LAPTM4B in OS. We identified miR-137 as a physiologically relevant regulator of LAPTM4B expression in OS. MiR-137 is capable of arranging cytoskeleton via targeting LAPTM4B. Moreover, the miR-137–LAPTM4B regulation further controls the cell migration in vitro and pulmonary metastasis in vivo. Mechanistically, LAPTM4B is essential in stimulating cytoskeleton organization through RhoA–LIMK–cofilin signaling axis, and LAPTM4B promotes RhoA protein stability via suppressing the ubiquitin-mediated proteasome degradation pathway. Our data in this study indicate miR-137–LAPTM4B axis represents a novel signal transduction pathway during the OS progression, which may be a visible target for cancer therapeutics development.

## Materials and methods

### Human cell lines and tissue specimens

The osteosarcoma cell line U2OS, MG-63, HOS, 143B and osteoblast hFOB1.19, were from American Type Culture Collection (ATCC). All the cells were authenticated by STR profiling, and were commonly tested for mycoplasma contamination.

LAPTM4B KO cells were generated by CRISPR/Cas9n as reported previously [22–24]. In Brief, coding sequences in exon 3 of LAPTM4B (Gene ID: 55353) were analyzed and selected for designing Cas9 nickase targets (http://crispr.mit.edu). We then subcloned Guide RNAs (sgRNAs) via BbsI sites. We further transfected cells with Cas9 nickase and sgRNA expressing plasmids, and selected cells with a culture medium containing puromycin (2 µg/ml) for 48 h. Subsequently, we isolated single clones and verified them via Sanger sequencing and western blotting.

For generating LAPTM4B stable overexpressing cell line, we transfected KO cells/ WT cells with pEFIRES-P containing LAPTM4B-24-3xFlag or CD63-3xFlag, using Lipofectamine LTX with PLUS reagent. Cells were grown in a culture medium containing 2 µg/ml puromycin until a resistant cell pool was formed. All osteosarcoma cells and osteoblast cell hFOB1.19 were maintained in DMEM + 10%FBS + L-glutamine + penicillin/ streptomycin, at 37 °C and with 5% CO_2_.

Osteosarcoma cancer tissues came from 31 patients undergoing resection. No patients had undergone treatment before the surgery. The tumor samples were from primary tumors. All of these samples were diagnosed according to the World Health Organization’s classification. Patrial samples from 10 patients were embedded in Paraffin, used for hematoxylin and Eosin (H&E) staining and Immunohistochemistry (IHC) staining; the rest part of the samples from 21 patients were snap-freeze in liquid nitrogen, used for qPCR and western blotting. This study was approved by the local research ethics committee, and written informed content from all patients were obtained following the instructions from the Declaration of Helsinki.

### Reagents, plasmids, antibodies, and siRNAs

Alexa Fluor 568 Phalloidin was from Molecular Probes (Cat#A-12380), Hiperfect transfection reagent was from QIAGEN (Cat#301705). Lipofectamine LTX with PLUS reagent was from Invitrogen (Cat#15338100). LAPTM4B-3′UTR luciferase plasmid (Cat#HmiT014362-MT01) and its negative Ctrl (Cat#CmiT000001-MT01) were purchased from GeneCopoeia, both plasmids were cloned from pEZX-MT01 vector. Agomir-137-3p (Cat#miR40000429-4-5) and the relative control were synthesized and purified by RiboBio.

The mouse monoclonal anti-Flag (M2) was from Sigma-Aldrich (Cat#F1804), mouse monoclonal anti-LAPTM4B from Atlas Antibodies (Cat#AMAb91356). Phospho-Myosin Light chain 2 antibody from Cell Signaling Technology (Cat#3671), Non-muscle Myosin Heavy Chain II-A antibody from Covance (Cat#PRB-440P), Cofilin (Phospho S3) antibody from Abcam (Cat#ab12866), Cofilin antibody from Abcam (Cat#ab42824), Phospho-LIMK1(Thr508)/LIMK2(Thr505) antibody from Cell Signaling Technology (Cat#3841), Flag antibody from Sigma-Aldrich (Cat#F1804), and Phospho-PAK antibody from Cell Signaling Technology (Cat#2601S). E-cadherin antibody from Proteintech (Cat#20874-1-AP), Vimentin antibody from ZENBIO (Cat#R22775), Phospho-AKT antibody from Cell Signaling Technology (Cat#4060), MMP2 antibody from Santa Cruz (Cat#sc-13595), and HA antibody from Proteintech (Cat#51064-2-AP).

RhoA antibody from Santa-Cruz (Cat#sc-418, used for immunoprecipitation and immunofluorescence) and Proteintech (Cat#66733-1-lg, used for western blotting). Protein A/G agarose beads from Santa Cruz (Cat#sc-2003), Ubiquitin antibody from Proteintech (Cat#10201-2-AP). GAPDH antibody from Proteintect (Cat#60004-1-lg), α-tubulin antibody from Proteintect (Cat#66031-1-lg).

The secondary antibodies Goat Anti-Mouse IgG (H + L)-HRP (Cat#1706516) and Goat Anti-Rat IgG (H + L)-HRP (Cat#1706515) were from BioRad. Cross-adsorbed Alexa Fluor 488 Goat Anti-Mouse (Cat#A-11001) was from ThermoFisher Scientific. The pre-designed “Silencer Select” LAPTM4B siRNA1 (GGAUCAGUAUAACUUUUCATT), LAPTM4B siRNA2 (CCUACCUGUUUGGUCCUUATT), and Ctrl siRNA were from Ambion.

### Q-PCR

For mRNA expression assay, total RNA was isolated from cells or tissues using RNA isolater Total RNA Extraction Reagent (Vazyme, Cat#R401-01). The cDNA was synthesized by Hifair®V one-step RT-gDNA digestion SuperMix for qPCR (Yeasen, Cat#11141ES60) using the StepOne Real-Time PCR System (Applied Biosystems, Foster City, CA, USA).

For miRNA expression assay, total RNA was reverse-transcribed into cDNA using the Hifair® III 1st Strand cDNA Synthesis Kit (YEASEN, Cat#11139ES10), and the qRT-PCR analysis was performed using the Hieff® qPCR SYBR Green Master Mix (YEASEN, Cat#11203ES03) according to the manufacturer’s instructions. All primers in our research were listed in Supplementary Table S1.

### In vivo metastasis assay

All Xenograft tumor experiments were performed in accordance with the regulations on animal experimentation and approved by the ethics committee of Anhui Medical University (Approval number: 20200490).

Female BALB/c nude mice (4 weeks) were purchased from Vital River Laboratories (Beijing, China) and housed under standard conditions. The animals were randomly divided into different groups.

In one experiment, the mice were implanted intravenously with U2OS cells or MG-63 cells (1 × 10^6^–6 × 10^6^ cells in 0.2 ml PBS) into the lateral tail vein, respectively. These mice were sacrificed six weeks after the injection.

In another experiment, 20 mice (five mice per group) were implanted intravenously with 143B cells (WT, CD63-Flag stably expressing, or LAPTM4B-Flag stably expressing), and treated intraperitoneally with 10 μmol/kg agomir-137-3p or the control once every three days for consecutive 4 weeks. The mice were sacrificed afterward and the pulmonary metastatic nodules in different groups were examined histologically, counted, and statistically analyzed. In the animal experiment, the investigators were not blinded to the group allocation during the experiment.

### Data mining

TARGET database (https://ocg.cancer.gov/programs/target) and Gene Expression Omnibus (https://www.ncbi.nlm.nih.gov/geo/) were employed to investigate the LAPTM4B expression in osteosarcoma. The CCLE database (https://sites.broadinstitute.org/ccle/) was used to assess the expression, copy number, and methylation state of LAPTM4B in the OS cell lines. The methylation percentage of LAPTM4B promoter in OS tumor tissues and control normal tissues were achieved from EWAS Data Hub (https://ngdc.cncb.ac.cn/ewas/datahub/index). TCGA and GTEx database were combined to investigate the relationship between LAPTM4B expression and cancer patients’ survival probability. To evaluate the expression levels of miRNAs and LAPTM4B, we used the python API “xenaPython”, to programmatically access data in the public Xena Data (Pancan Atlax Hub). The co-expression/ physical interaction between LAPTM4B and RhoA was predicted by GeneMANIA (http://genemania.org/). GeneMANIA predicts gene function and the relation of input genes, via using a large number of functional association data including genomics and proteomics data, protein and genetic interactions, co-expression, co-localization, protein domain similarity, and so on [34].

### Statistical analysis

All the data are presented as the mean ± standard error of the mean (SEM) from at least three independent experiments. Statistical significance was calculated using the Student’s t-test for pairwise comparisons, and Holm’s *t*-test for multiple comparisons. The relationship between the miRNA levels and gene expression was determined using Pearson’s correlation coefficient test. The level of statistical significance was set at 0.05. *p < 0.05.

Additional detailed descriptions of methods are in Supplemental Materials and Methods.

## Results

### Expression of LAPTM4B is elevated in OS

**Fig. 1:**
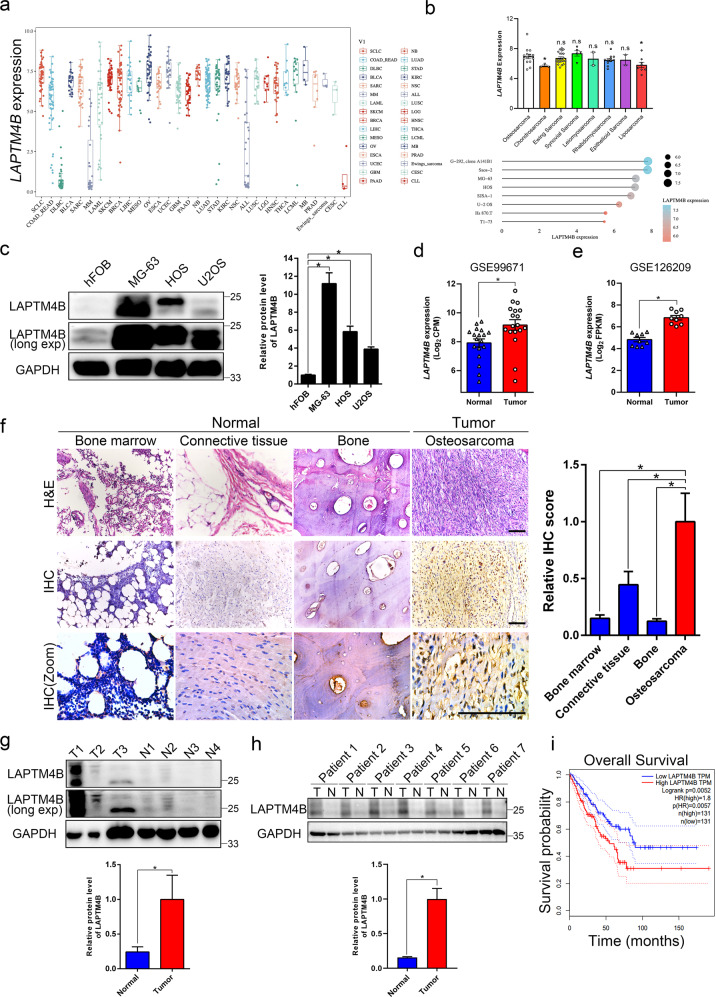
Upregulation of LAPTM4B associated with survival probability in OS patients. **a** The detailed LAPTM4B mRNA expression in different cancer-type cell lines from the CCLE database. **b** The LAPTM4B mRNA expression in OS cell lines. Upper panel: LAPTM4B expression in different lineage subtypes of sarcoma cell lines, *p*(Osteosarcoma, Chondrosarcoma) = 0.04, *p*(Osteosarcoma, Liposarcoma) = 0.015. Down panel: the detailed expression level in several representative OS cells. *p*-value was calculated by comparing expression data in each individual group with expression data in “Osteosarcoma”. * denotes “*p* < 0.05”, “n.s” denotes “no significance”. **c** The protein levels of LAPTM4B in hFOB1.19, MG-63, HOS, and U2OS were measured by western blotting. Left panel: representative experiment. Right panel: quantification of *n* = 3 experiments, mean ± SEM, data normalized to “hFOB1.19”, *p*(hFOB, MG-63) = 0.0145, *p*(hFOB, HOS) = 0.0368, *p*(hFOB, U2OS) = 0.0226. “long exp”: “long exposure”. **d** The LAPTM4B expression (Log_2_CPM) in OS tumor tissue and control normal tissue from the GEO database (GSE99671). Mean ± SEM, *p*(Normal, Tumor)=0.008. “Normal tissues” represent “Bone tissue”. **e** The LAPTM4B expression (Log_2_FPKM) in OS tumor tissue and control normal tissue from the GEO database (GSE126209). Mean ± SEM, *p*(Normal, Tumor) = 0.0005. “Normal tissues” represent “Adjacent normal tissue”. **f** The LAPTM4B protein levels in tumor tissue and adjacent normal tissues from collected OS surgery samples were determined by immunohistochemistry. Left panel: representative images from H&E staining and IHC staining. Right panel: quantification of IHC results. Data from 127 images from 10 patients. Mean ± SEM, data normalized to “Osteosarcoma”, *p*(Osteosarcoma, Bone marrow) = 0.0125, *p*(Osteosarcoma, Connective tissue) = 0.0344, *p*(Osteosarcoma, Bone) = 0.0112. Scale bar: 0.125 mm. **g** The LAPTM4B protein levels in tumor tissue and adjacent normal tissue from collected OS surgery samples were determined by western blotting. Upper panel: representative experiment. Down panel: quantification of *n* = 3 technical repeats, mean ± SEM, data normalized to “Normal”, *p*(Normal, Tumor) = 0.0014. T tumor tissue, N adjacent normal tissue. **h** The LAPTM4B protein levels in tumor and paired-normal tissue from seven OS patients were determined by Western blotting. Upper panel: representative experiment. Down panel: quantification of *n* = 3 technical repeats, mean ± SEM, data normalized to “Normal”, *p*(Normal, Tumor) = 0.000245. T tumor tissue, N adjacent normal tissue. **i** The overall survival probability of sarcoma patients in terms of LAPTM4B expression from the combined analysis from TCGA and GTEx database.

In order to assess the functional roles of LAPTM4B in OS, we first mined the Cancer Cell Line Encyclopedia (CCLE) database and found LAPTM4B expression in sarcoma is relatively high among other cells (Fig. 1a and Supplementary Fig. S1a). Sarcoma includes OS, chondrosarcoma, Ewing sarcoma, synovial sarcoma, leiomyosarcoma, and so on, the detailed expression of LAPTM4B in these cell lines was analyzed (Fig. 1b and Supplementary Table S2). We next examined LAPTM4B protein level in cultured cells, and found that LAPTM4B was elevated in OS cell lines compared with osteoblast cell line hFOB1.19 (Fig. 1c).

Analyzing data from the Gene Expression Omnibus (GEO) database revealed that LAPTM4B was upregulated in OS tumor tissues compared with normal control tissues (GSE99671, GSE126209) (Fig. 1d, e). Elevated LAPTM4B expression was further observed in surgery tissue samples collected from OS patients via both immunohistochemistry and immunoblotting (Fig. 1f, g). Additionally, we examined LAPTM4B levels in tumor and paired-normal tissue from seven patients, the results confirmed LAPTM4B is upregulated in OS tumor tissue (Fig. 1h).

We next investigated the clinical relevance of increased LAPTM4B expression. Combined analysis from TCGA and GTEx databases indicated that LAPTM4B expression negatively correlates with overall survival expectation in both sarcoma (Fig. 1i) and pan-cancer including 33 types of cancer (Supplementary Fig. S1b). Further analysis from GEO (GSE16102) and TARGET database showed LAPTM4B expression negatively corelates with the overall survival in OS (Supplementary Fig. S1c). The expression level and clinical relevance suggest that the upregulated LAPTM4B may be involved in the OS progression with crucial functions.

### LAPTM4B is essential for stress fiber organization

**Fig. 2:**
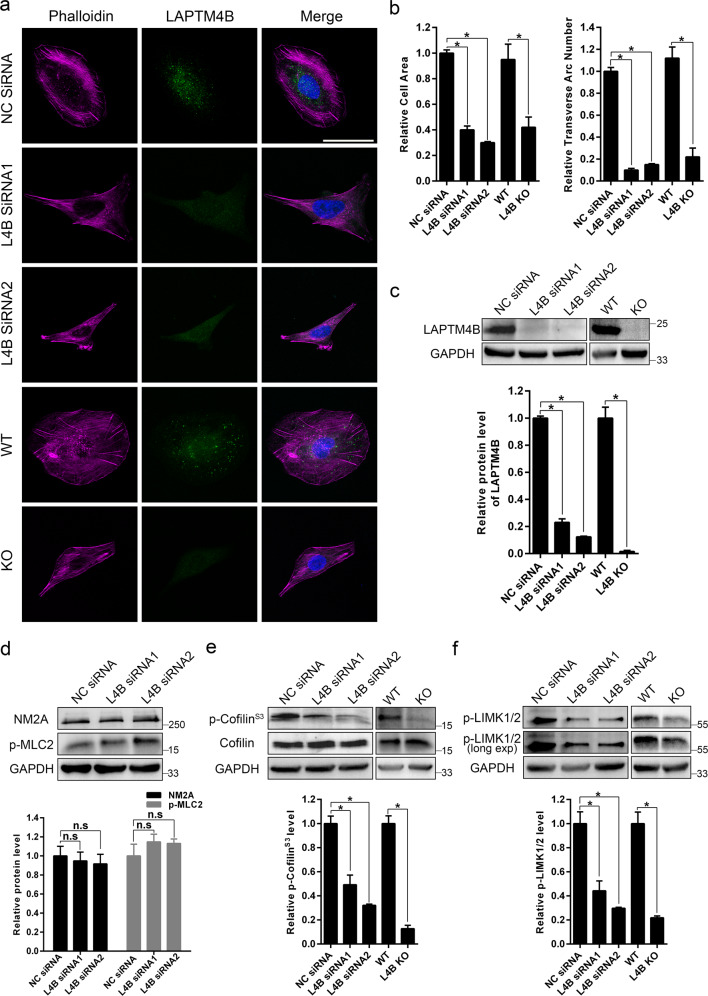
LAPTM4B is essential for stress fiber organization. **a** Stress fibers in U2OS cells were visualized by phalloidin staining in the indicated cells. Scale bar: 50 µm. For siRNA-treated cells, 72 h after transfection, cells were fixed for immunofluorescence. **b** Cell area (left panel) and transverse arc numbers (right panel) in the indicated cells were quantified. Quantification of *n* = 3 experiments, *n* > 53 cells per each group. Mean ± SEM, data normalized to “NC siRNA” or “WT”. For cell area, *p*(NC siRNA, L4B siRNA1) = 0.0354, *p*(NC siRNA, L4B siRNA2) = 0.0238, *p*(WT, L4B KO) = 0.0395. For transverse arc numbers, *p*(NC siRNA, L4B siRNA1)=0.0093, *p*(NC siRNA, L4B siRNA2)=0.0126, *p*(WT, L4B KO) = 0.0189. WT wide type, L4B LAPTM4B. **c** LAPTM4B protein levels were determined by Western blotting. Upper panel: representative experiment. Down panel: quantification of *n* = 3 experiments, mean ± SEM, data normalized to “NC siRNA” or “WT”, *p*(NC siRNA, L4B siRNA1) = 0.0138, *p*(NC siRNA, L4B siRNA2) = 0.0076, *p*(WT, L4B KO) = 0.001. **d** The NM2A protein level and MLC2 phosphorylation level were determined by western blotting in U2OS cells with the indicated treatment. Upper panel: representative experiment. Down panel: quantification of *n* = 3 experiments, mean ± SEM, data normalized to “NC siRNA”. For siRNA-treated cells, 72 h after transfection, cells were harvested for Western blotting. **e** The total protein level and phosphorylation level (Phospho S3) of Cofilin were determined by western blotting in the indicated cells. Upper panel: representative experiment. Down panel: quantification of *n* = 3 experiments, mean ± SEM, data normalized to “NC siRNA” or “WT”, *p*(NC siRNA, L4B siRNA1) = 0.0256, *p*(NC siRNA, L4B siRNA2)=0.0134, *p*(WT, L4B KO) = 0.008. **f** The phosphorylation level of LIMK1/2 was determined by Western blotting in the indicated cells. Upper panel: representative experiment. Down panel: quantification of *n* = 3 experiments, mean ± SEM, data normalized to “NC siRNA” or “WT”, *p*(NC siRNA, L4B siRNA1)=0.0351, *p*(NC siRNA, L4B siRNA2) = 0.0242, *p*(WT, L4B KO) = 0.0213.

Due to the elevated LAPTM4B expression in OS and well-established functions in other cancers, we next investigated LAPTM4B roles in OS. Intriguingly, silencing of LAPTM4B in U2OS cells by using two distinct siRNAs induced a similar reshaping of cell morphology and stress fiber pattern, as stained by phalloidin (Fig. 2a). Further quantification displayed a significant reduction of cell area and transverse arc numbers after LAPTM4B downregulation (Fig. 2b). Importantly, the alterations were also observed in LAPTM4B knockout (KO) U2OS cells generated by CRISPR-Cas9n (Fig. 2a–c), suggesting LAPTM4B displays an essential regulatory role in stress fiber organization.

Stress fibers have vital roles in cellular activities central to tumor progression, e.g. cell proliferation, and cell movement [5]. We therefore examined whether the major machineries involved in stress fiber dynamics were affected by LAPTM4B. Phosphorylation of myosin light chain (MLC) has been reported to be necessary for stress fiber assembly [35], and cofilin phosphorylation at Ser3 inactivates its actin-binding capability, thereby promoting stress fiber disassembly [9]. We found that LAPTM4B depletion didn’t induce any significant difference in either non-muscle myosin 2 A (NM2A) levels or the phosphorylation state of MLC (Fig. 2d). Conversely, the phosphorylation of cofilin, a key regulator in stress fiber disassembly, is significantly decreased in LAPTM4B siRNA treated cells and in LAPTM4B KO cells, while the total cofilin amount is unaltered (Fig. 2e).

Since LIMK can phosphorylate cofilin at the Ser3 [9], we hypothesized that LAPTM4B provokes stress fiber arrangement via the LIMK-cofilin pathway. Indeed, p-LIMK levels were reduced in cells either by silencing or knockout of LAPTM4B (Fig. 2f).

### LAPTM4B promotes RhoA stability via suppressing the ubiquitin-proteasome degradation

**Fig. 3:**
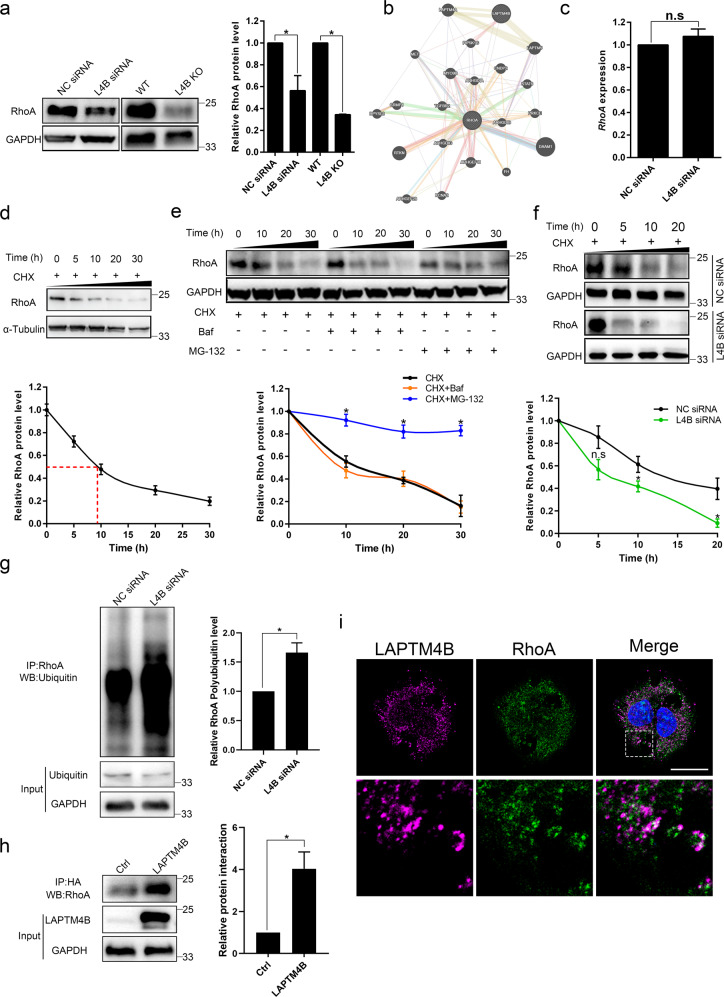
LAPTM4B suppresses the ubiquitin-proteasome degradation of RhoA. **a** The RhoA protein levels were measured by Western blotting in LAPTM4B depleted U2OS cells. Left panel: representative experiment. Right panel: quantification of *n* = 3 experiments, mean ± SEM, data normalized to “NC siRNA” or “WT”. *p*(NC siRNA, L4B siRNA) = 0.0243, *p*(WT, L4B KO) = 0.007. For siRNA-treated cells, 72 h after transfection, cells were harvested for Western blotting. **b** Bioinformatics prediction by GeneMANIA indicated the potential co-expression/ physical interaction between LAPTM4B and RhoA. **c** U2OS cells were transfected with indicated siRNAs, 48 h after transfection, the RhoA mRNA expression were measured by Q-PCR. **d**. U2OS cells were treated with 50 µg/mL cycloheximide (CHX) for the indicated times, and the RhoA protein levels were assessed by Western blotting. Upper panel: representative experiment. Down panel: quantification of *n* = 3 experiments, mean ± SEM. The red dashed line indicates the time point when half of the endogenous RhoA has been degraded. **e**. U2OS cells were treated with 1 µmol/L bafilomycin-A1 (Baf) or 20 µmol/L MG-132, together with 50 µg/mL CHX for the indicated times, and the RhoA protein levels were assessed by Western blotting. Upper panel: representative experiment. Down panel: quantification of *n* = 3 experiments, mean ± SEM. *p < 0.05. **f** U2OS cells were transfected with indicated siRNA, the cells were then treated with 50 µg/mL CHX for the indicated times and the RhoA protein levels were assessed by western blotting. Upper panel: representative experiment. Down panel: quantification of *n* = 3 experiments, mean ± SEM. *p < 0.05. **g** U2OS cells were transfected with indicated siRNA and harvested for immunoprecipitation after 72 h, cells were treated with 20 µmol/L MG-132 for 9 h before the harvest. The immunoprecipitation was performed with RhoA antibody, the lysates afterwards were immunoblotted with antibody against Ubiquitin. Left panel: representative experiment. Right panel: quantification of *n* = 3 experiments, mean ± SEM, data normalized to “NC siRNA”. *p*(NC siRNA, L4B siRNA)=0.0231. **h** HA-tagged LAPTM4B stably expressing cells and the control cells were immunoprecipitated by HA antibody, the lysates were subsequently immunoblotted with antibody against RhoA. Left panel: representative experiment. Right panel: quantification of *n* = 3 experiments, mean ± SEM, data normalized to “Ctrl”. *p*(Ctrl, LAPTM4B) = 0.03687. **i** The immunofluorescence experiments were performed in HA-tagged LAPTM4B stably expressing cells via using anti-HA antibody (magenta) and anti-RhoA antibody (green). Scale bar: 20 µm. The region of a dashed white box is amplified in the down panel.

The phosphorylation of LIMK is under dynamic regulation by PAK (p21 protein-activated kinase), ROCK (Rho-associated protein kinase), and MRCK (myotonic dystrophy kinase-related Cdc42-binding kinase) [8]. These molecules can be activated by GTPases, *e.g*. Cdc42 and Rac regulate PAK [36], Cdc42 actives MRCK [37], whilst RhoA/ROCK/LIMK cascade plays crucial roles in stress fiber formation and OS development [8]. We therefore questioned whether these key proteins are regulated by LAPTM4B. Western blotting reports no difference with the phosphorylation level of PAK (Supplementary Fig. S2a, b), which is in agreement with our recent finding that Cdc42 (the upstream GTPase of PAK1/2) activity was not adjusted by LAPTM4B [24]. We further investigated whether LAPTM4B displays any regulatory effect on RhoA. Interestingly, the RhoA protein level was significantly reduced in LAPTM4B depleted U2OS cells (Fig. 3a), a similar reduction was found in another OS cell line MG-63 (Supplementary Fig. S3), which is in line with the bioinformatically predicted potential co-expression/ physical interaction between LAPTM4B and RhoA (Fig. 3b). In this GeneMANIA prediction analyzing the functional relation between LAPTM4B and RhoA [34], the network weight index of predicted physical interaction (between proteins) suggests LAPTM4B may interact with RhoA protein.

We then investigated the detailed mechanism underpinning LAPMT4B regulation of RhoA. Q-PCR experiments reported no difference regarding the RhoA transcript levels (Fig. 3c), suggesting LAPTM4B may instead regulate the stability of RhoA protein. We next treated cells with cycloheximide to inhibit protein synthesis, our data revealed that RhoA contains an ~10 h half-life in U2OS cells (Fig. 3d).

To further dissect the key pathways involved in the RhoA degradation, U2OS cells were incubated with the V-ATPase inhibitor bafilomycin-A1 to suppress the autophagy/lysosomal degradation pathway, or with MG-132 to block the proteasome degradation. Our data show that MG-132 treatment, but not bafilomycin-A1, can significantly attenuate the protein degradation in cycloheximide treated cells (Fig. 3e), suggesting proteasome system is the major degradation pathway of RhoA in U2OS cells, in agreement with the previous report in MLE12 cells [38]. We then investigated whether the proteasome degradation pathway of RhoA was regulated by LAPTM4B, intriguingly, LAPTM4B depletion enhances the degradation of RhoA in cycloheximide treated cells (Fig. 3f), suggesting LAPTM4B can stabilize RhoA protein by inhibiting its proteasome degradation. We further assessed the effect of LAPTM4B on RhoA ubiquitination via immunoprecipitation by RhoA antibody and subsequently measuring the ubiquitin level. As expected, LAPTM4B was found to suppress RhoA ubiquitination (Fig. 3g).

To exclude the possibility of cell-specific effect, we next utilized another OS cell line 143B for further experiments. The stress fiber organization was altered in LAPTM4B KO 143B cells, quantification showed the stress fiber number and cell area were significantly reduced by the downregulation of LAPTM4B (Supplementary Fig. S4a). Moreover, RhoA, as well as the phosphorylation of LIMK and cofilin, were downregulated in LAPTM4B KO 143B cells (Supplementary Fig. S4b). Importantly, the ubiquitination level of RhoA was suppressed by LAPTM4B in 143B cells (Supplementary Fig. S4c). These results indicated LAPTM4B displays the vital regulatory function of stress fiber in OS cells.

We next questioned whether LAPTM4B interacted with RhoA. To this end, we performed immunoprecipitation experiments via pulling down by HA antibody in HA-tagged LAPTM4B stably expressing cells, and subsequently measuring the RhoA level by western blotting. Our results found RhoA was significantly enriched in LAPTM4B stably expressing cells, compared with in the control cells (Fig. 3h). The immunofluorescence experiments further observed the partial co-localization between LAPTM4B and RhoA (Fig. 3i). We anticipated the interaction may be the potential mechanism underlying LAPTM4B regulatory function of RhoA stability.

### Low expression of miR-128 and miR-137 induces the LAPTM4B upregulation

**Fig. 4:**
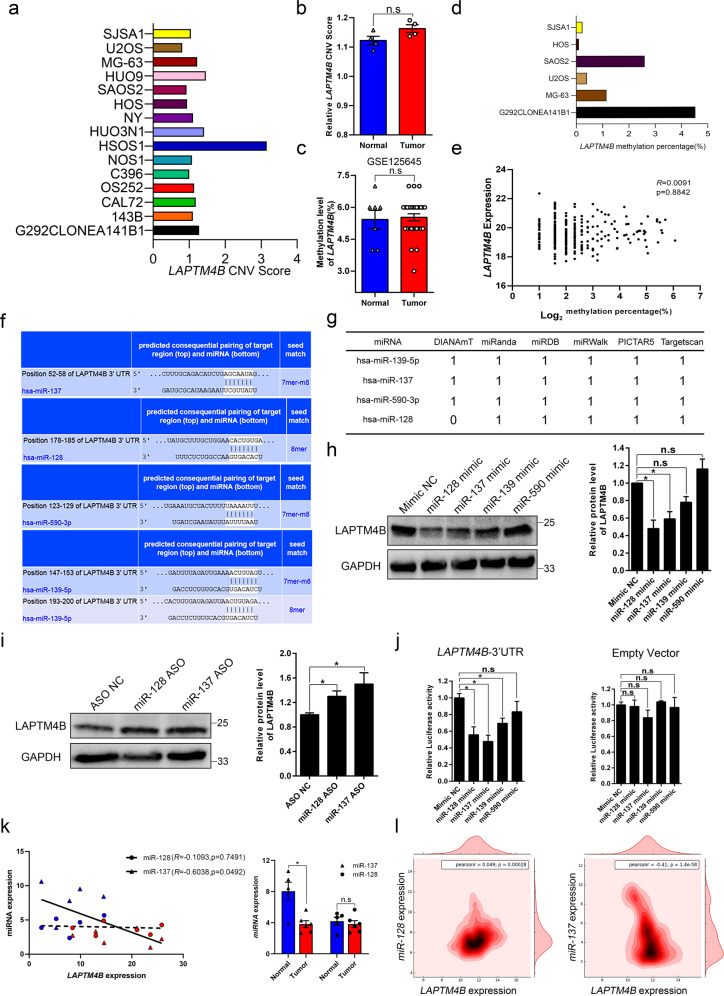
miRNAs induce the elevated expression of LAPTM4B. **a** The LAPTM4B copy number score (CNV score) in different OS cell lines from the CCLE database. **b** The LAPTM4B copy number score in collected OS tumor tissue samples and adjacent normal tissue samples. **c** The methylation percentage of LAPTM4B in OS tumor tissue and control normal tissue from the GEO database (GSE125645, probe cg22581831). **d** The methylation percentage of LAPTM4B in representative OS cell lines from the CCLE database. **e** The correlation between LAPTM4B expression and methylation percentage in tissue samples (OS tissues and normal control tissues) from analyzing data in EWAS Data Hub. **f** Bioinformatics prediction of miRNAs targeting LAPTM4B by TargetScan. **g** Prediction of miRNAs targeting LAPTM4B by other bioinformatics tools. “1” represents “positive prediction”, “0” represents “negative prediction”. **h** U2OS cells were treated with the indicated miRNA mimics, 72 h after transfection, LAPTM4B protein levels were assessed by western blotting. Left panel: representative experiment. Right panel: quantification of *n* = 3 experiments, mean ± SEM, data normalized to “Mimic NC”, *p*(Mimic NC, miR-128 mimic) = 0.0239, *p*(Mimic NC, miR-137 mimic) = 0.0341. **i** U2OS cells were treated with the indicated miRNA ASO, 72 h after transfection, LAPTM4B protein levels were assessed by western blotting. Left panel: representative experiment. Right panel: quantification of *n* = 3 experiments, mean ± SEM, data normalized to “ASO NC”, *p*(ASO NC, miR-128 ASO) = 0.0457, *p*(ASO NC, miR-137 ASO) = 0.0386. **j** Dual-luciferase assay indicates miRNAs directly targeting the 3′UTR of LAPTM4B. Left Panel: Cells were co-transfected with LAPTM4B-3′UTR and different miRNA mimics, 32 h after transfection, relative luciferase activity was measured afterwards. Quantification of *n* = 3 experiments, mean ± SEM, data normalized to “Mimic NC”, *p*(Mimic NC, miR-128 mimic) = 0.0374, *p*(Mimic NC, miR-137 mimic) = 0.0211, *p*(Mimic NC, miR-139 mimic) = 0.0405. Right panel: relative luciferase activity after co-transfection with empty vector and different miRNA mimics. **k**. The expression level of miR-128, miR-137, and LAPTM4B in collected OS surgery samples were determined by q-PCR. The red color represents “OS tumor tissue”, blue color represents “adjacent normal tissue”. Left panel: correlation analysis of miR-137 and LAPTM4B mRNA levels (solid line, Pearson’s method, *R* = − 0.6038, *p* = 0.03656), miR-128 and LAPTM4B mRNA levels (dotted line, Pearson’s method, *R* = − 0.1093, *p* = 0.7491). Right panel: The expression of miR-128 and miR-137 in OS tumor tissue (Tumor) and adjacent normal tissue (Normal). For miR-137, *p*(Tumor, Normal) = 0.0008. **l**. The expression level of miR-128, miR-137, and LAPTM4B in tissue samples (tumors tissue and normal control tissue) were achieved from the TCGA database and GTEx database.

Due to the significantly elevated expression of LAPTM4B in OS and its function in cytoskeleton arrangement, we next investigated the molecular mechanisms underpinning LAPTM4B dysregulation. As most cancer cells undergo genome instability and gene amplification [27], we examined the copy number variation (CNV) in the CCLE database and found OS cell lines do not display increased LAPTM4B copy numbers (Fig. 4a), which was supported by the data that LAPTM4B CNV doesn’t display any significant shift in collected OS tumor samples compared with normal control tissues (Fig. 4b).

DNA methylation acts as a major epigenetic regulation in cancers [29]. We analyzed the data from GEO database (GSE125645, GSE161407) and EWAS data hub, and found that LAPTM4B methylation percentage is not significantly altered in OS tumor tissues compared with normal control tissues (Fig. 4c, Supplementary Fig. S5a-c). This notion was further supported by the CCLE database that no meaningful methylation within the LAPTM4B promoter region was identified among OS cell lines (Fig. 4d). Moreover, data analysis from EWAS data hub indicates LAPTM4B expression displays no significant correlation with the methylation state in OS tumor tissues and control normal tissues (Fig. 4e). These data suggest that DNA methylation unlikely induces the dysregulation of LAPTM4B in OS.

As CNV and DNA methylation status of LAPTM4B appear unaltered in OS, we next questioned whether the post-transcriptional regulation, e.g. miRNA, was involved. MiRNAs suppress gene expression by directly binding the “seed sequences” in the 3’ Untranslated Region (UTR) [33]. We conducted bioinformatics analysis of potential miRNAs targeting LAPTM4B. In silico analysis by TargetScan revealed four most potential candidates (Fig. 4f), which were simultaneously predicted by other bioinformatics tools, i.e. DIANAmT, miRanda, miRDB, miRWalk, and PICTAR5 (Fig. 4g).

To determine whether these miRNAs downregulate LAPTM4B expression, we transfected cells with miRNAs mimics and found that miR-128/ miR-137 mimics induced a substantial reduction in LAPTM4B protein levels (Fig. 4h), we next transfected cells with miRNA antisense oligonucleotides (ASO) and found inhibiting of either miR-128 or miR-137 moderately increased LAPTM4B levels (Fig. 4i). These data indicate that miR-128 and miR-137 can downregulate LAPTM4B protein levels. To further validate whether miRNAs directly bind to the LAPTM4B mRNA 3’UTR, we conducted a dual luciferase assay by co-transfecting LAPTM4B 3’UTR luciferase vector with miRNA mimics. Our data showed that miR-128, miR-137, and miR-139 mimics transfection induced a significant reduction of the relative luciferase activity (Fig. 4j), but no drop was observed in luciferase activity when co-transfecting with the empty vector (Fig. 4j), indicating that these miRNAs directly bind to the LAPTM4B mRNA 3’UTR. Together, our experiments by western blotting and dual luciferase assay confirmed that miR-128 and miR-137 directly target LAPTM4B and modulate the protein expression.

In the collected clinical OS samples, miR-137 but not miR-128 negatively correlated with LAPTM4B expression. Moreover, miR-137 was downregulated in OS tumor samples (Fig. 4k), which is in line with previous reports that miR-137 acts as one of the most downregulated miRNAs in OS [39, 40]. As the number of our collected tissue samples is limited, we next conducted data mining to explore the relationship from more samples. By a combined analysis of TCGA and GTEx database, we found a significantly negative correlation between miR-137 and LAPTM4B (Fig. 4l). However, miR-128 displays no meaningful correlation with LAPTM4B (Fig. 4l).

These data suggest that miRNAs, rather than DNA CNV or methylation status, contribute to the LAPTM4B upregulation in OS, and miR-137 targeting LAPTM4B could be involved in OS cancer progression.

### The regulation of miR-137 on stress fiber is LAPTM4B-dependent

**Fig. 5:**
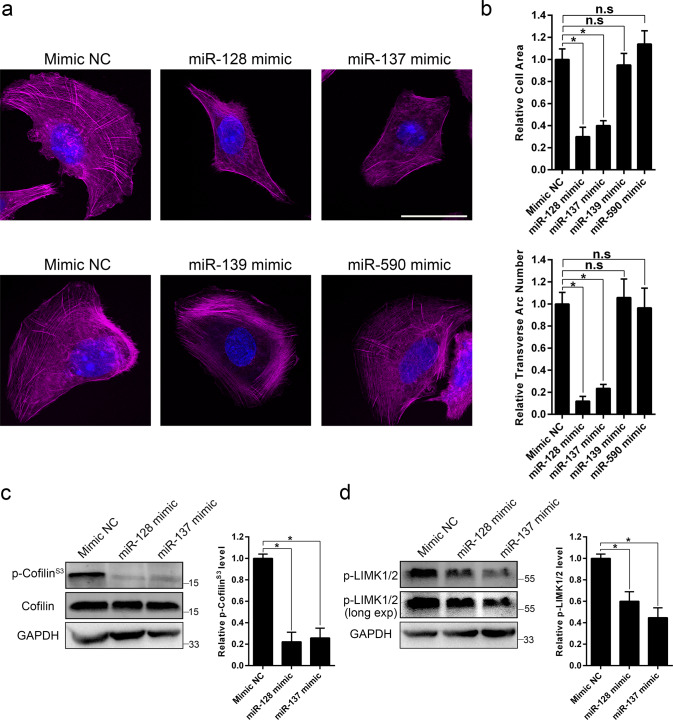
miR-128 and miR-137 regulate stress fiber arrangement. **a** U2OS cells were treated with the indicated miRNA mimics, 72 h after transfection, stress fibers were visualized by phalloidin staining. Scale bar: 50 µm. **b** Cell area (Upper panel) and transverse arc number (Down panel) in the indicated cells were quantified. Quantification of *n* = 3 experiments, *n* > 46 cells per each group. Mean ± SEM, data normalized to “Mimic NC”. For cell area, *p*(Mimic NC, miR-128 mimic) = 0.0125, *p*(Mimic NC, miR-137 mimic) = 0.0341. For transverse arc number, *p*(Mimic NC, miR-128 mimic) = 0.0082, *p*(Mimic NC, miR-137 mimic) = 0.0114. **c** The total protein and phosphorylation level (Phospho S3) of cofilin in U2OS cells transfected by indicated miRNA mimics. Cells were harvested for western blotting 72 h after transfection. Left panel: representative experiment. Right panel: quantification of *n* = 3 experiments, mean ± SEM, data normalized to “Mimic NC”, *p*(Mimic NC, miR-128 mimic) = 0.0162, *p*(Mimic NC, miR-137 mimic) = 0.0243. **d** The phosphorylated LIMK1/2 in U2OS cells transfected by indicated miRNA mimics. Left panel: representative experiment. Right panel: quantification of *n* = 3 experiments, mean ± SEM, data normalized to “Mimic NC”, *p*(Mimic NC, miR-128 mimic) = 0.0342, *p*(Mimic NC, miR-137 mimic) = 0.0224.

Since miR-128 and miR-137 directly target LAPTM4B and regulate the expression, we herein questioned whether these two miRNAs display any effect on stress fiber. To this end, we transfected cells with miRNA mimics, and the phalloidin staining reported significantly distinct stress fiber pattern, reduced cell area and transverse arc numbers after overexpressing miR-128 and miR-137, but not miR-139 and miR-590 (Fig. 5a, b). We then measured the phosphorylation levels of cofilin and LIMK, our data showed that both miR-128 and miR-137 overexpression reduced the amount of phosphorylated cofilin (Fig. 5c) and phosphorylated LIMK (Fig. 5d).

**Fig. 6:**
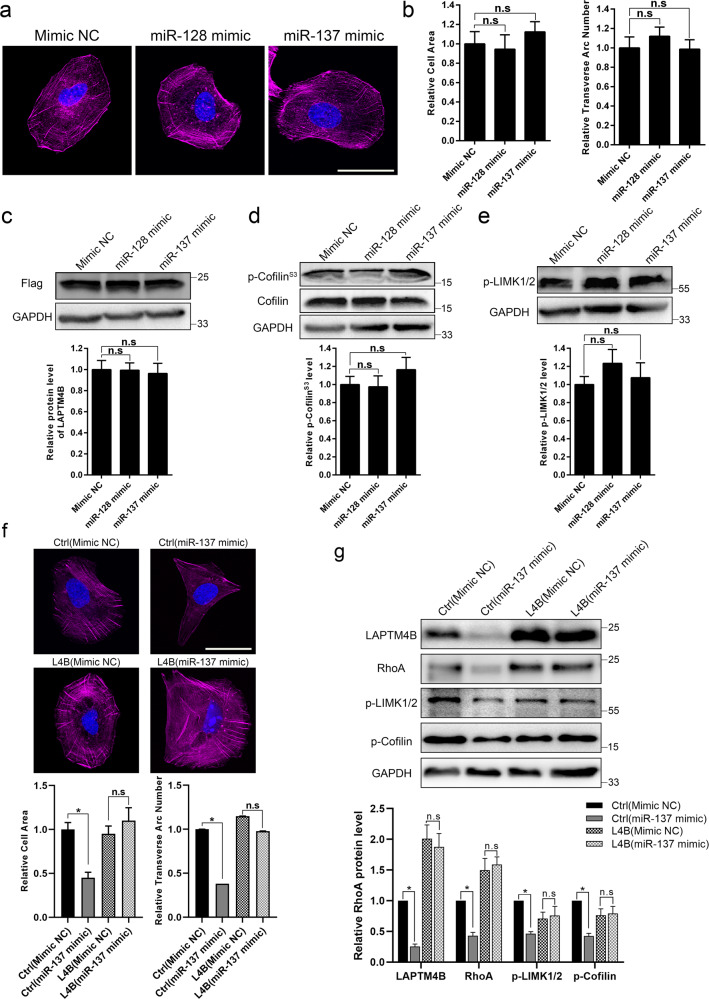
The regulation of miR-137 on stress fiber is LAPTM4B-dependent. **a** LAPTM4B stably expressing U2OS cells were treated with the indicated miRNA mimics, 72 h after transfection, stress fibers were visualized by phalloidin staining. Scale bar: 50 µm. **b** Cell area (Left panel) and transverse arc number (Right panel) in the indicated cells were quantified. Quantification of *n* = 3 experiments, *n* > 42 cells per each group. Mean ± SEM, data normalized to “Mimic NC”. **c** LAPTM4B stably expressing U2OS cells transfected by indicated miRNA mimics, 72 h after transfection, the LAPTM4B levels were then determined by western blotting with anti-Flag antibody. Up panel: representative experiment. Down panel: quantification of *n* = 3 experiments, mean ± SEM, data normalized to “Mimic NC”. **d** The total protein and phosphorylation level (Phospho S3) of cofilin in LAPTM4B stably expressing U2OS cells transfected by indicated miRNA mimics. Up panel: representative experiment. Down panel: quantification of *n* = 3 experiments, mean ± SEM, data normalized to “Mimic NC”. **e** LAPTM4B stably expressing U2OS cells transfected by indicated miRNA mimics, the phosphorylated LIMK1/2 was then determined by western blotting. Up panel: representative experiment. Down panel: quantification of *n* = 3 experiments, mean ± SEM, data normalized to “Mimic NC”. **f** LAPTM4B stably expressing U2OS cells from WT background and the control cells were treated with the Mimic NC or miR-137 mimic. 72 h after transfection, stress fibers were visualized by phalloidin staining. Scale bar: 50 µm. Upper panel: representative images. Down panel: Cell area and transverse arc number in the indicated cells were quantified. Quantification of *n* = 3 experiments, *n* > 31 cells per each group. Mean ± SEM, data normalized to “Ctrl(Mimic NC)”. **g** LAPTM4B stably expressing U2OS cells from WT background and the control cells were treated with the Mimic NC or miR-137 mimic. Cells were harvested for Western blotting 72 h after transfection. Up panel: representative experiment. Down panel: quantification of *n* = 3 experiments, mean ± SEM, data normalized to “Ctrl(Mimic NC)”.

One specific miRNA is capable of targeting multiple transcripts and thus is involved in regulating various cellular functions [33]. We therefore asked whether these miRNAs’ effects on stress fiber are dependent on LAPTM4B, but not via other targets. To this end, LAPTM4B stably expressing U2OS cells which lacks the 3′UTR of LAPTM4B were generated from KO background. We didn’t document any considerable shift regarding cell morphology and stress fiber pattern after transfecting miR-128/ miR-137 mimics in LAPTM4B stably expressing cells (Fig. 6a–c), suggesting that these miRNAs’ function in regulating cytoskeleton are mediated through LAPTM4B. Accordingly, the phosphorylation level of cofilin and LIMK were not altered by miRNAs in LAPTM4B stably expressing U2OS cells (Fig. 6d, e).

Due to the significantly negative correlation between miR-137 and LAPTM4B in collected OS samples (Fig. 4k), we next focus on miR-137–LAPTM4B functional roles during OS procession. To further confirm that the miR-137–LAPTM4B signal axis is indeed essential for maintaining the stress fiber, we generated LAPTM4B stably expressing U2OS cells from the WT background. In control cells, miR-137 transfection is capable of inducing the dysregulated stress fiber (Fig. 6f), and the reduced levels of RhoA, p-LIMK, and p-cofilin were observed accordingly (Fig. 6g). However, miR-137 appears to have no effect on stress fiber organization and the related signaling pathway in LAPTM4B stably expressing U2OS cells (Fig. 6f, g).

Moreover, we generated LAPTM4B stably expressing 143B cells from the WT background. The impact of miR-137–LAPTM4B on the regulation of stress fiber as well as the related molecules can also be observed in 143B cells (Supplementary Fig. S6a, b). These experiments indicate that the miR-137–LAPTM4B axis displays a significant regulatory effect on stress fiber organization via the LIMK-cofilin signal pathway in OS cells.

### miR-137 suppresses OS cell migration and pulmonary metastasis via targeting LAPTM4B

miR-137 is one of the most downregulated miRNAs in OS [40], which was also observed in our collected OS tumor samples (Fig. 4k). Previous studies by us and others have revealed that LAPTM4B promotes cell migration and metastasis in various cancers [24, 41, 42]. We questioned whether LAPTM4B displayed a similar function in OS cells. Therefore, we examined the LAPTM4B effect on cell migration in OS cells via a label-free and real-time xCELLigence measurement system [43], our data showed depletion of LAPTM4B significantly attenuated the migratory capability of both U2OS and 143B cells (Supplementary Fig. S7a, b). Previous studies reported that LAPTM4B promotes cancer cell migration via regulating epithelial-mesenchymal transition (EMT) [44], phosphorylation of AKT [45], and the release of matrix metalloprotein (MMP) [46], we next examined whether these molecules were regulated by LAPTM4B in OS cells. Interestingly, no significant difference in these proteins was observed between LAPTM4B WT and KO OS cells (Supplementary Fig. S7c), suggesting the cytoskeleton arrangement could be a novel mechanism underlying LAPTM4B migration-stimulating functions in OS.

**Fig. 7:**
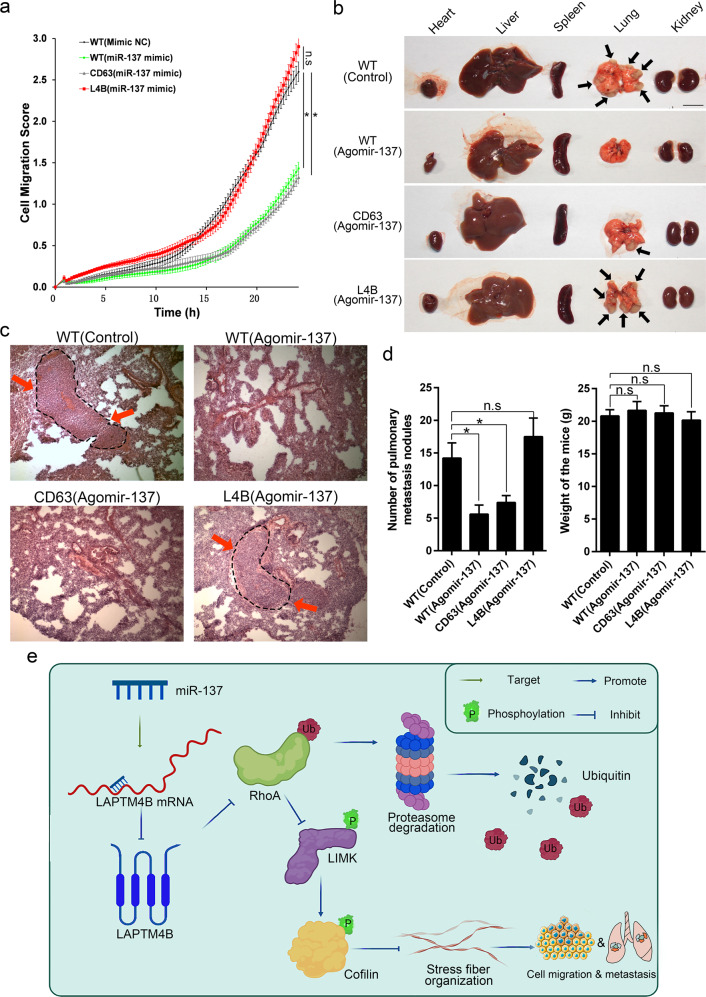
miR-137 inhibits cell migration and pulmonary metastasis via targeting LAPTM4B. **a** Real-time and lable-free measurement of cell migration in WT, LAPTM4B- or CD63-stably expressing U2OS cells transfected with miR-137 mimic or the Mimic NC, using the xCELLigence system. 6 × 10^4^ cells in serum-free medium were seeded in the top chamber, the cell migration index was measured at an interval of 15 min for consecutive 25 h. More than 12 wells for each group from four independent experiments were analyzed, mean ± SEM. **b** 20 mice (five mice per group) were implanted intravenously with 143B cells (WT, CD63 stably expressing, or LAPTM4B stably expressing), and treated intraperitoneally with 10 μmol/kg agomir-137-3p or the control once every three days for consecutive 4 weeks. The mice were sacrificed afterward, and organs were imaged for visualizing the metastasis nodules. Arrow: metastatic nodules. Scale bar: 1 cm. **c** Representative histopathological images of lung sections. Arrow: the metastatic tumor. **d** Data analysis of different parameters in this experiment. Left: Number of pulmonary metastasis nodules, *p*(WT_Control, WT_Agomir-137) = 0.0375, *p*(WT_Control, CD63_Agomir-137) = 0.0412; Right: weight of the mice (g) in different groups. **e** Schematic diagram depicting the LAPTM4B function and mechanism in OS. LAPTM4B, which is specifically inhibited by miR-137, promotes RhoA stability via suppressing the ubiquitin-mediated proteasome degradation, the stabilized RhoA protein subsequently promotes stress fiber organization through the LIMK-cofilin pathway. In this manner, LAPTM4B stimulates cell migration and cancer metastasis.

We hypothesized that miR-137 may inhibit cell migration and cancer metastasis via targeting LAPTM4B. To this end, we examined the cell migration in miR-137 mimic transfected WT or LAPTM4B stably expressing cells. Our data revealed that miR-137 mimic treatment, but not the Mimic NC, significantly inhibited the migratory capability of U2OS cells. Moreover, overexpressing LAPTM4B but not the control protein CD63 could rescue this phenotype (Fig. 7a). Similar results were obtained by wound-healing experiments (Supplementary Fig. S8a, b). Together, these data indicate that miR-137 suppresses OS cell migration via targeting LAPTM4B.

To further gain in vivo insight into miR-137–LAPTM4B role in cancer metastasis, we employed the nude mice xenograft model. A series range of 1 × 10^6^–6 × 10^6^ U2OS cells or MG-63 cells were injected into the tail vein of BABL/c female nude mice. The mice were sacrificed six weeks after the injection, however, no metastatic nodule was observed (Supplementary Fig. S9a, b). Since we could not obtain the necessary in vivo data by using U2OS and MG-63 cells, we instead employed 143B, which is reported and utilized in studying lung metastasis [47]. In this experiment, the mice were firstly implanted intravenously with 4 × 10^6^ 143B cells and followed by miRNA treatment, our data showed that the mice intraperitoneally injected with agomir-137-3p displayed a significantly smaller number of visible metastatic nodules when compared with control mimic (Fig. 7b). Interestingly, the metastatic-suppressing effect of agomir-137-3p was not observed in mice implanted with LAPTM4B stably expressing 143B cells, but can still be documented in mice implanted with CD63 stably expressing 143B cells (Fig. 7b). The metastases were further verified by histological examination **(**Fig. 7c**)**, further statistical analysis indicated that miR-137 inhibits the pulmonary metastasis via targeting LAPTM4B (Fig. 7d).

In summary, our study identified the high expression and the clinical relevance of LAPTM4B in OS. We established that miR-137 targets LAPTM4B, regulates stress fiber organization, further suppresses OS cell migration and pulmonary metastasis. The effects are mediated by the dysregulated RhoA-LIMK-cofilin signaling pathway. Moreover, we found that LAPTM4B stabilizes RhoA protein via suppressing the ubiquitin-proteasome degradation pathway (Fig. 7e). The correlation between miR-137 and LAPTM4B expression in OS patient tissue samples and cancer databases, together with the regulatory functions in cytoskeleton arrangement, cancer cell migration and pulmonary metastasis, underscore the clinical significance of the current discovery.

## Discussion

Despite great advances have occurred in the molecular profiling of OS over last decades, the current standard treatment is largely resection combined with chemotherapies. Deciphering the molecular mechanisms underlying OS progression and metastasis is urgently required for developing targeted therapies and improving patients’ survival probability. The cytoskeleton is essential in regulating cell migration and cancer metastasis, which is the leading cause of OS patients’ death. In the current study, we uncovered that LAPTM4B regulates stress fiber organization and cell morphology via the RhoA-LIMK-cofilin pathway, which could be a novel target for OS therapy development.

An intriguing discovery in this study is the non-canonical function of LAPTM4B in the cytoskeleton arrangement. To the best of our knowledge, this is the first study to investigate the lysosomal protein LAPTM4B function in stress fiber organization. Our data uncovered that LAPTM4B suppresses the proteasomal degradation of RhoA and promotes the downstream LIMK-cofilin signaling. The potential protein interaction between LAPTM4B and RhoA was confirmed by immunoprecipitation and immunofluorescence. We anticipated this interaction may account for the underlying LAPTM4B regulatory function of RhoA stability. However, the exact mechanism and the interacting motifs are still open and worthy of further investigation. This study provides a scenario that the lysosomal protein may participate in cytoskeleton organization via regulating the proteasome degradation or autophagy/lysosome degradation of certain key proteins, and further affects cell movement and eventual patients’ survival probability.

Previous studies have shown that LAPTM4B promotes cancer cell migration via epithelial-mesenchymal transition (EMT) [44], phosphorylation of AKT [45], and the release of matrix metalloprotein (MMP) [46], we recently found that LAPTM4B displays partial filopodia distribution, promoting cancer cell migration via stimulating integrin beta1 recycling and focal adhesion dynamics [24]. Here in this study, we uncovered another molecular mechanism involved, *i.e*. LAPTM4B can enhance cell migration and cancer metastasis via regulating the cytoskeleton arrangement in OS.

Elevated expression of LAPTM4B has been showed in cancers [11, 45]. However, the underlying molecular mechanisms are not sufficiently understood, albeit several transcriptional regulations have been reported [48, 49]. LAPTM4B gene localized in chromosome 8q.22.1, a region containing the MYC oncogene and is amplified in breast cancer [50] and prostate cancer [51], which prompted us to investigate whether the LAPTM4B copy number is increased in OS. Surprisingly, LAPTM4B copy number was not amplified in OS. Meanwhile, the methylation status of the LAPTM4B promoter region appeared not altered in OS. A previous study by Zhang et al. found that miR-188 downregulates LAPTM4B and modulates prostate cancer development [15]. Here in this study, we established that miR-137 binds to LAPTM4B 3’UTR and downregulates the expression, providing new mechanical insight into LAPTM4B dysregulation in cancers. Moreover, the observed low expression of miR-137 in OS tumor samples, together with the functional assays in vitro and in vivo, indicate that this miRNA displays an instrumental regulatory role in migration and metastasis via controlling LAPTM4B expression. These findings may serve as one rationale for future OS targeted therapeutics development.

Even though our data from clinical OS patients’ samples and cancer databases support the findings from cells and the xenograft model, other methodologies including the PDX model or transgenic mice (e.g. LAPTM4B KO mice) were warranty to illustrate LAPTM4B functions and roles in osteosarcoma progression in the future study.

## Supplementary information

Supplementary information

Supplemental Materials and Methods

Supplementary Table 2

## Data Availability

All the data generated during the current study are available from the corresponding author on reasonable request.
