# Supplementary material for: miR-137–LAPTM4B regulates cytoskeleton organization and cancer metastasis via the RhoA-LIMK-Cofilin pathway in osteosarcoma
Source: Oncogenesis. 2023 May 6;12(1):25. doi: 10.1038/s41389-023-00471-5 (PMC10163001; doi:10.1038/s41389-023-00471-5)
Supplement: Supplementary file 1 — Supplementary information [file 41389_2023_471_MOESM1_ESM.docx]

**miR-137–LAPTM4B regulates cytoskeleton organization and cancer metastasis via the RhoA-LIMK-Cofilin pathway in osteosarcoma**

Ruyu Yan^1,#^, Dan Liu^1,#^, Junjie Wang^1^, Minxia Liu^1,2^, Hongjuan Guo^1^, Jing Bai^1^, Shuo Yang^3^,Jun Chang^3^, Zhihong Yao^4^, Zuozhang Yang^4^, Tomas Blom^5,6*^, Kecheng Zhou^1,5,6*^

^1^ School of Life Sciences, Anhui Medical University, Hefei, 230032, China

^2^ Institute for Molecular Medicine Finland, Helsinki Institute of Life Science, University of Helsinki, Helsinki, 00290, Finland

^3^ Department of Orthopaedics, The First Affiliated Hospital of Anhui Medical University, Hefei, 230032, China

^4^ Bone and Soft Tissue Tumours Research Centre of Yunnan Province, Department of Orthopaedics, The Third Affiliated Hospital of Kunming Medical University (Yunnan Cancer Hospital, Yunnan Cancer Center), Kunming, Yunnan, 650118, China

^5^ Department of Anatomy, Faculty of Medicine, University of Helsinki, Helsinki, 00014, Finland

^6^ Minerva Foundation Institute for Medical Research, Helsinki, 00014, Finland

^#^ Ruyu Yan and Dan Liu contribue equally to the current study

*Corresponding author

Prof. Dr. Kecheng Zhou

Group Leader of “Cancer Metabolism Laboratory”

School of Life Science, Anhui Medical University

81 Meishan Road, 230032, Hefei, China

E-mail: zhoukecheng@ahmu.edu.cn

Dr. Tomas Blom

Department of Anatomy

Faculty of Medicine, University of Helsinki

Haartmaninkatu 8, 00014, Helsinki, Finland

E-mail: tomas.blom@helsinki.fi

**Supplemental Information**

**
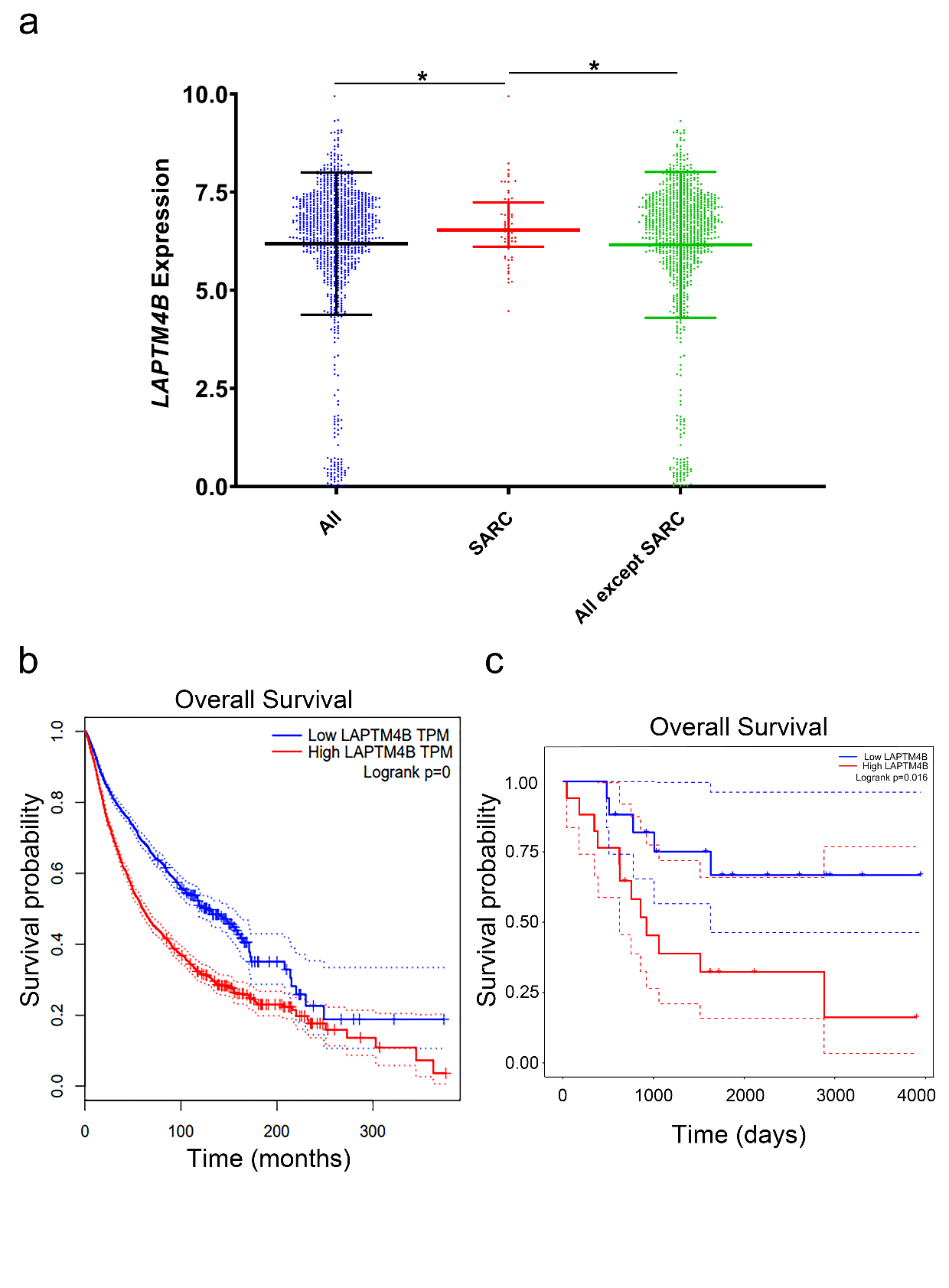
**

**Supplementary Figure S1. LAPTM4B expression correlated with survival expectations in cancer patients**

1. Analysis from the CCLE database indicates LAPTM4B expression in sarcoma is significantly higher than the average expression in other cells. “SARC”: “sarcoma cell lines”; “All”: All the cell lines listed in the CCLE database; “All except SARC”: All the other cell lines except sarcoma cell lines. p(All, SARC)= 0.0276, p(SARC, All except SARC)= 0.023.
2. The survival probability of pan-cancer patients in terms of LAPTM4B expression from a combined analysis of TCGA and GTEx database. Pan-cancer includes 33 types of cancer.
3. The survival probability of osteosarcoma patients in terms of LAPTM4B expression from a combined analysis of GEO database (GSE16102) and TARGET database. n=34.


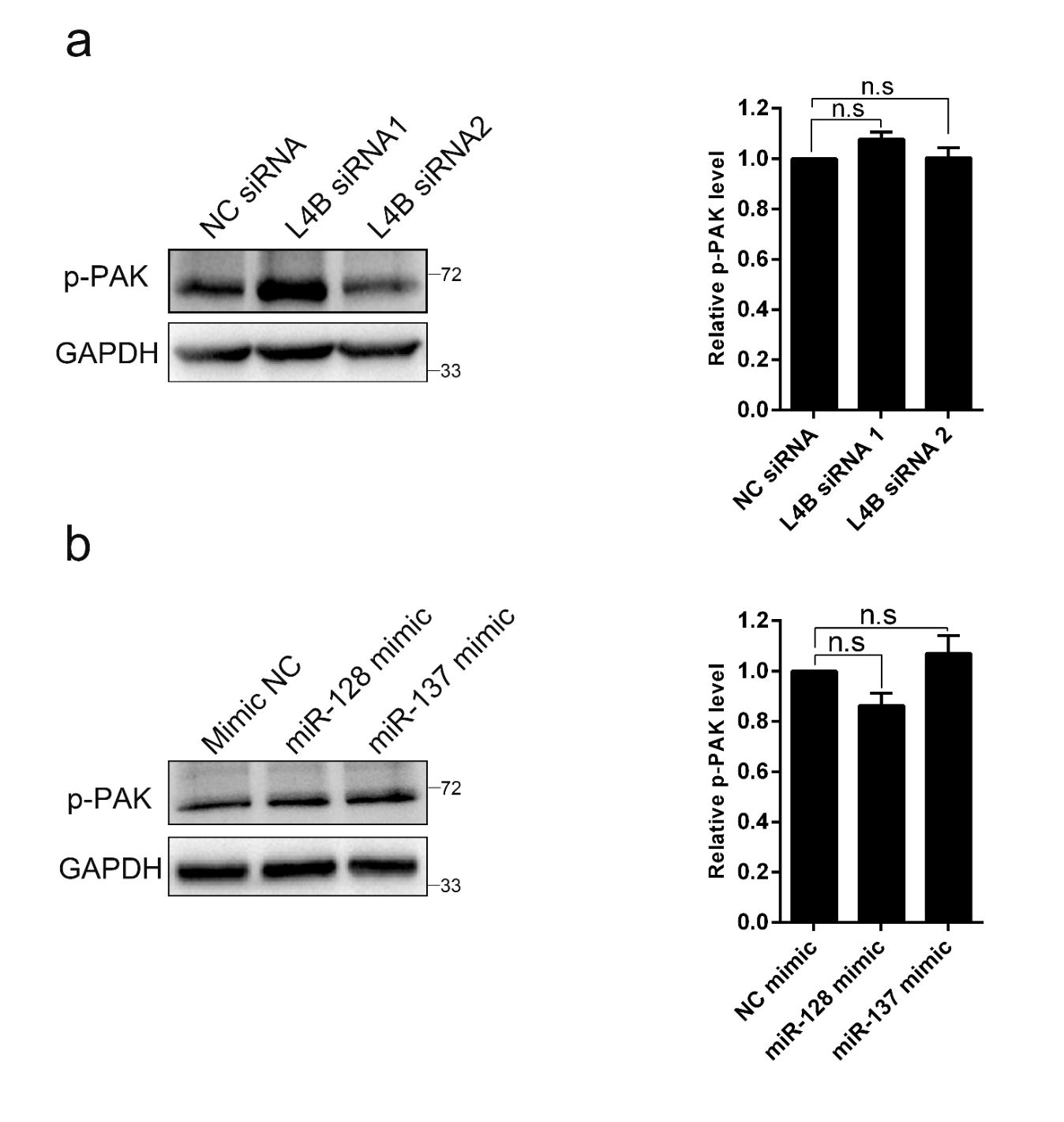


**Supplementary Figure S2. The phosphorylation level of PAK not altered by LAPTM4B, miR-128 or miR-137**

1. U2OS cells were transfected by indicated siRNA, 72 h after transfection, the phosphorylated PAK level was then determined by western blotting. Left panel: representative experiment. Right panel: quantification of n=3 experiments, mean ± SEM, data normalized to “NC siRNA”.
2. U2OS cells were transfected by indicated miRNA mimics, 72 h after transfection, the phosphorylated PAK level was then determined by western blotting. Left panel: representative experiment. Right panel: quantification of n=3 experiments, mean ± SEM, data normalized to “Mimic NC”.


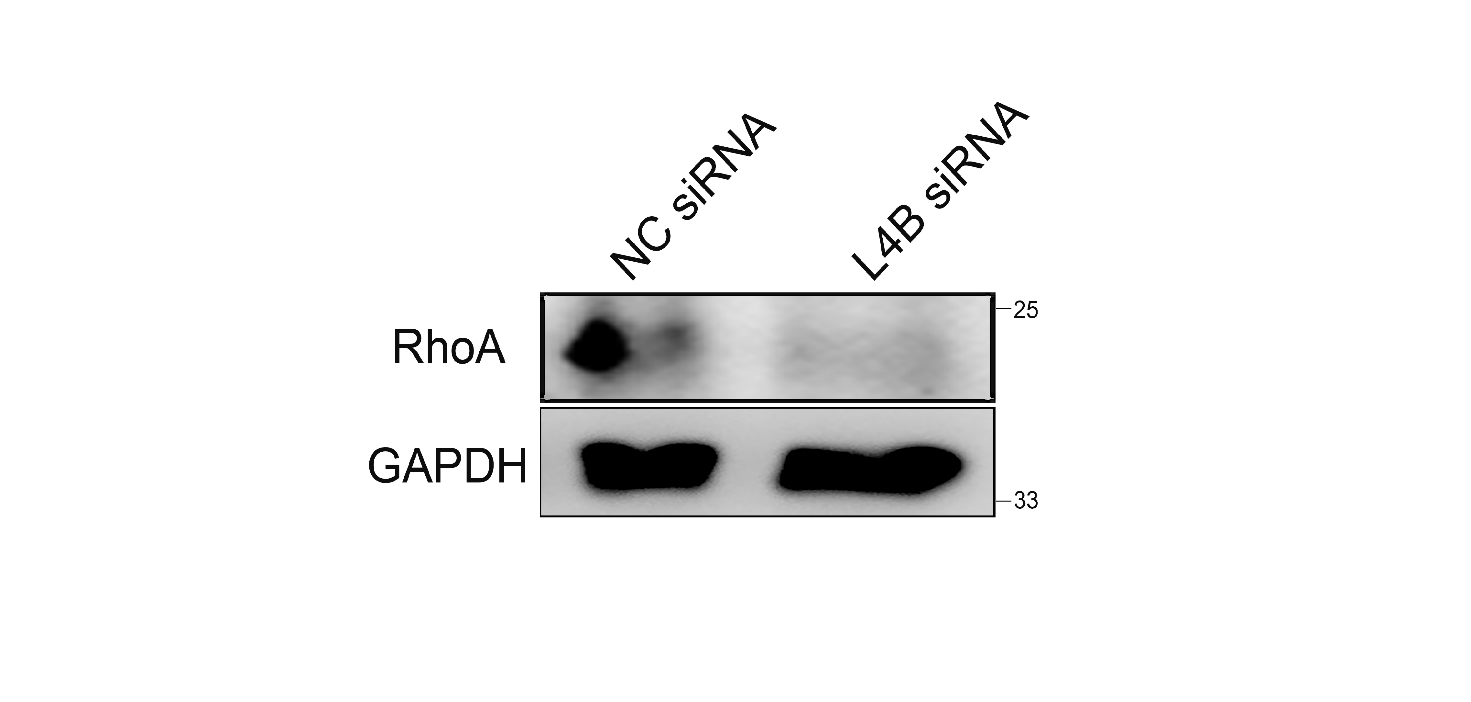


**Supplementary Figure S3. LAPTM4B upregulates RhoA protein level in MG-63 cells**

MG-63 cells were transfected by indicated siRNA, 72 h after transfection, the RhoA levels were then determined by Western blotting.


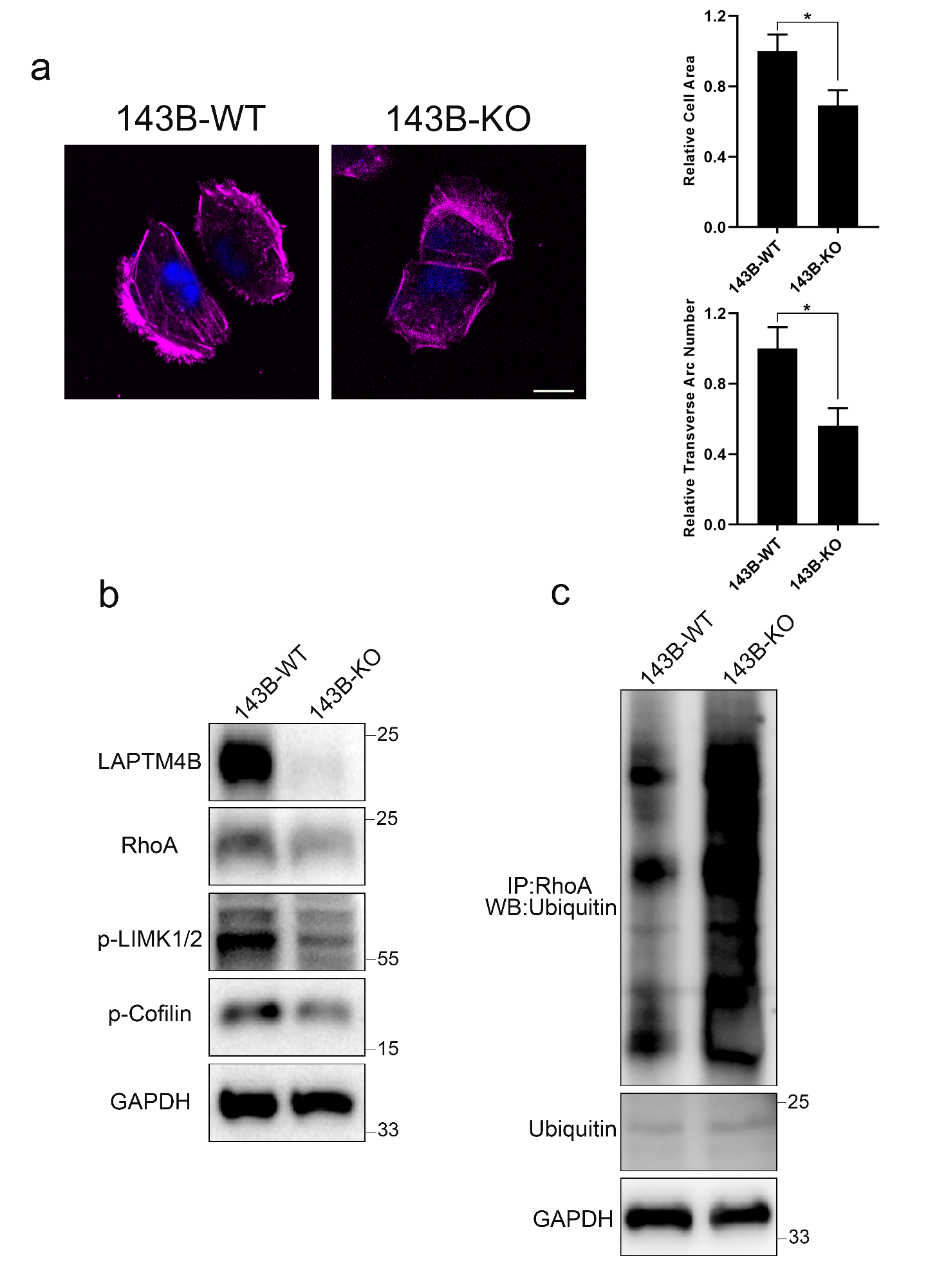


**Supplementary Figure S4. LAPTM4B regulates stress fiber organization and related signal pathway in 143B cells**

1. Stress fibers in WT and LAPTM4B KO 143B cells were visualized by phalloidin staining. Scale bar: 20 µm. Left panel: representative images. Right panel: Cell area and transverse arc number in the indicated cells were quantified. Quantification of n=3 experiments, n > 24 cells per each group. Mean ± SEM, data normalized to “143B-WT”.
2. The protein levels of LAPTM4B, RhoA, p-LIMK1/2, and p-Cofilin in WT and LAPTM4B KO 143B cells were determined by Western blotting.
3. WT and LAPTM4B KO 143B cells were treated with 20 µmol/L MG-132 for 9 h, and harvested for immunoprecipitation. The immunoprecipitation was performed with RhoA antibody, the lysates afterwards were immunoblotted with antibody against Ubiquitin.


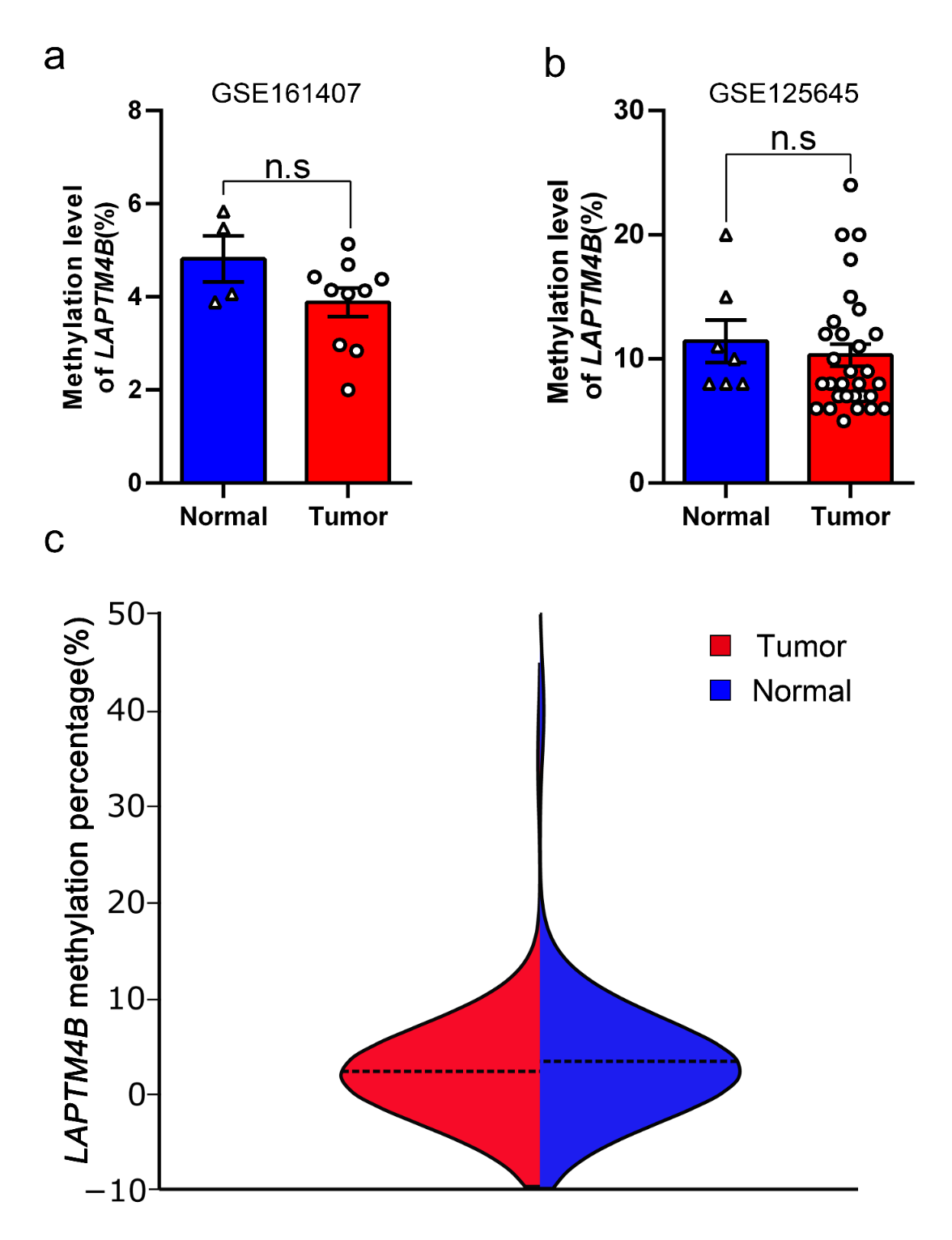


**Supplementary Figure S5. LAPTM4B methylation percentage not altered in OS**

1. The methylation percentage of LAPTM4B in OS tumor tissue and control normal tissue from the GEO database (GSE161407).
2. The methylation percentage of LAPTM4B in OS tumor tissue and control normal tissue from the GEO database (GSE125645, probe cg20541656).
3. The methylation percentage of LAPTM4B promoter in OS tumor tissue and control normal tissue from the data analysis in EWAS Data Hub.


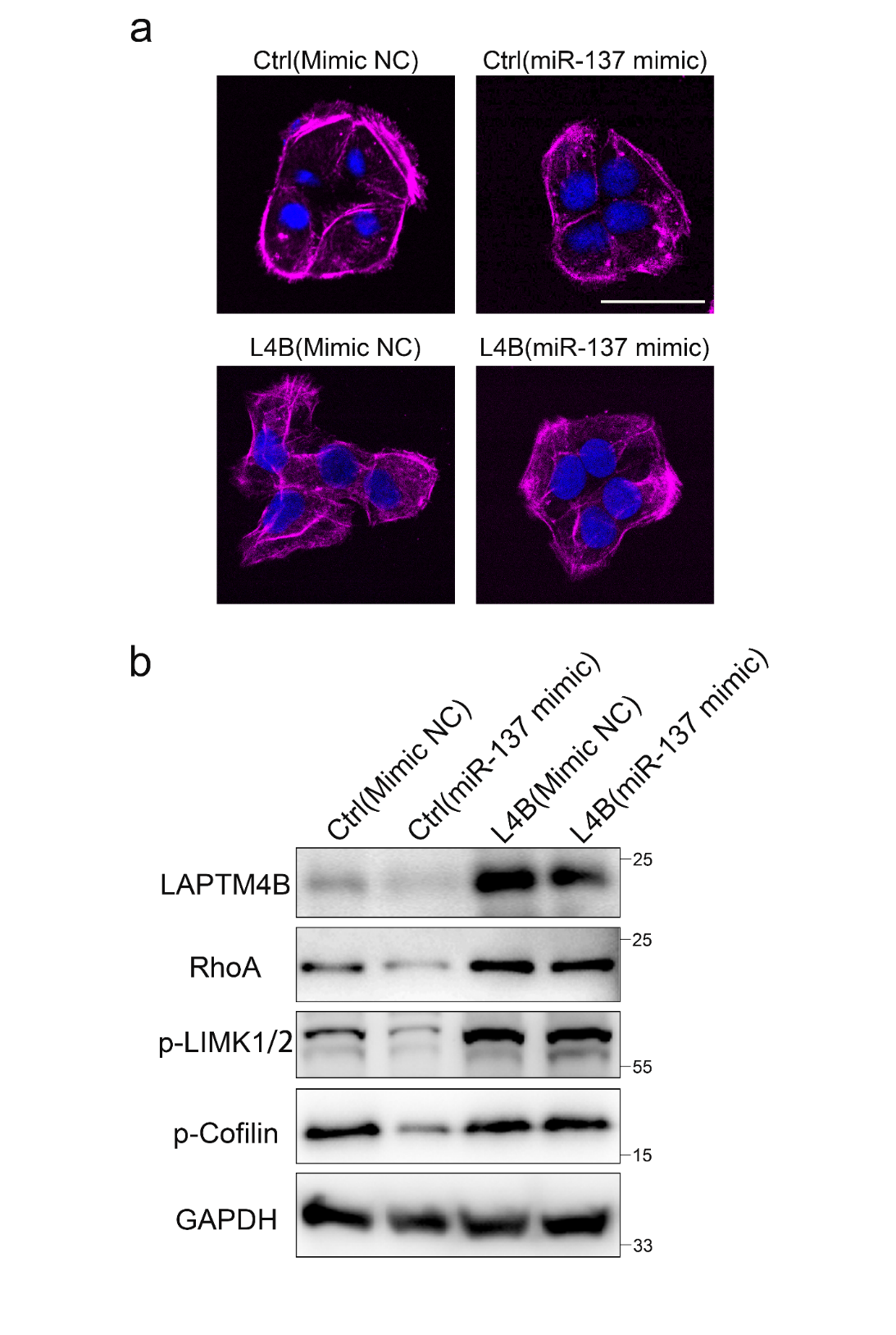


**Supplementary Figure S6. miR-137–LAPTM4B axis displays a significant regulatory effect on stress fiber organization in 143B cells**

1. LAPTM4B stably expressing 143B cells and the control cells were treated with the Mimic NC or miR-137 mimic. 72 h after transfection, stress fibers were visualized by phalloidin staining. Scale bar: 50 µm.
2. LAPTM4B stably expressing 143B cells from WT background and the control cells were treated with the Mimic NC or miR-137 mimic. Cells were harvested for Western blotting 72 h after transfection.


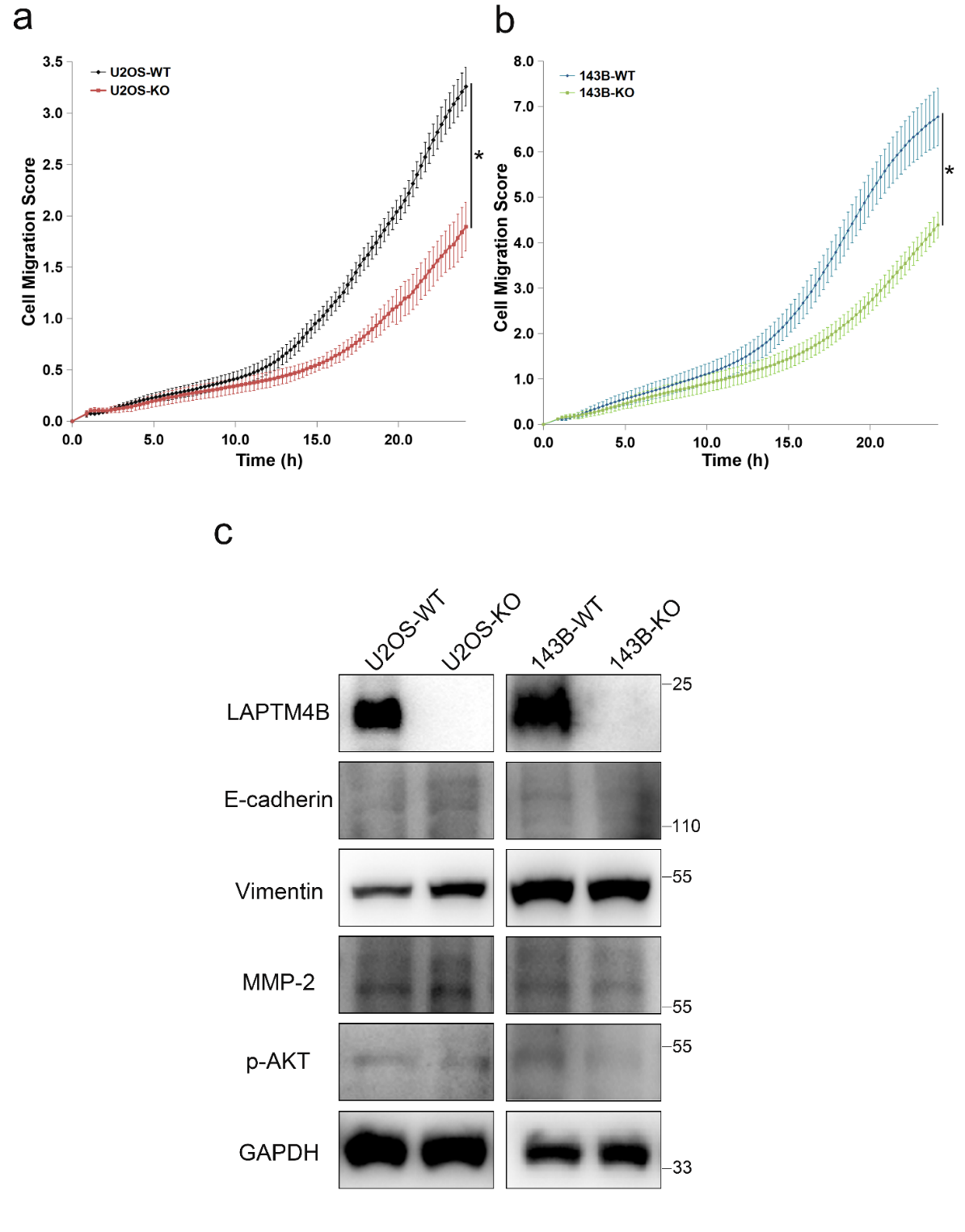


**Supplementary Figure S7. LAPTM4B upregulates the migratory capability of OS cells.**

1. Cell migration in WT, LAPTM4B KO U2OS cells was measured by the xCELLigence system. 6×10^4^ cells in serum-free medium were seeded in the top chamber, the cell migration index was measured at an interval of 15 min for a consecutive 25 h. More than 9 wells for each group from three independent experiments were analyzed, mean ± SEM.
2. Cell migration in WT, LAPTM4B KO 143B cells was measured by the xCELLigence system. mean ± SEM.
3. The expression of E-cadherin, Vimentin, MMP-2, and the phosphorylated AKT in WT and LAPTM4B KO OS cells were determined by Western blotting.

**
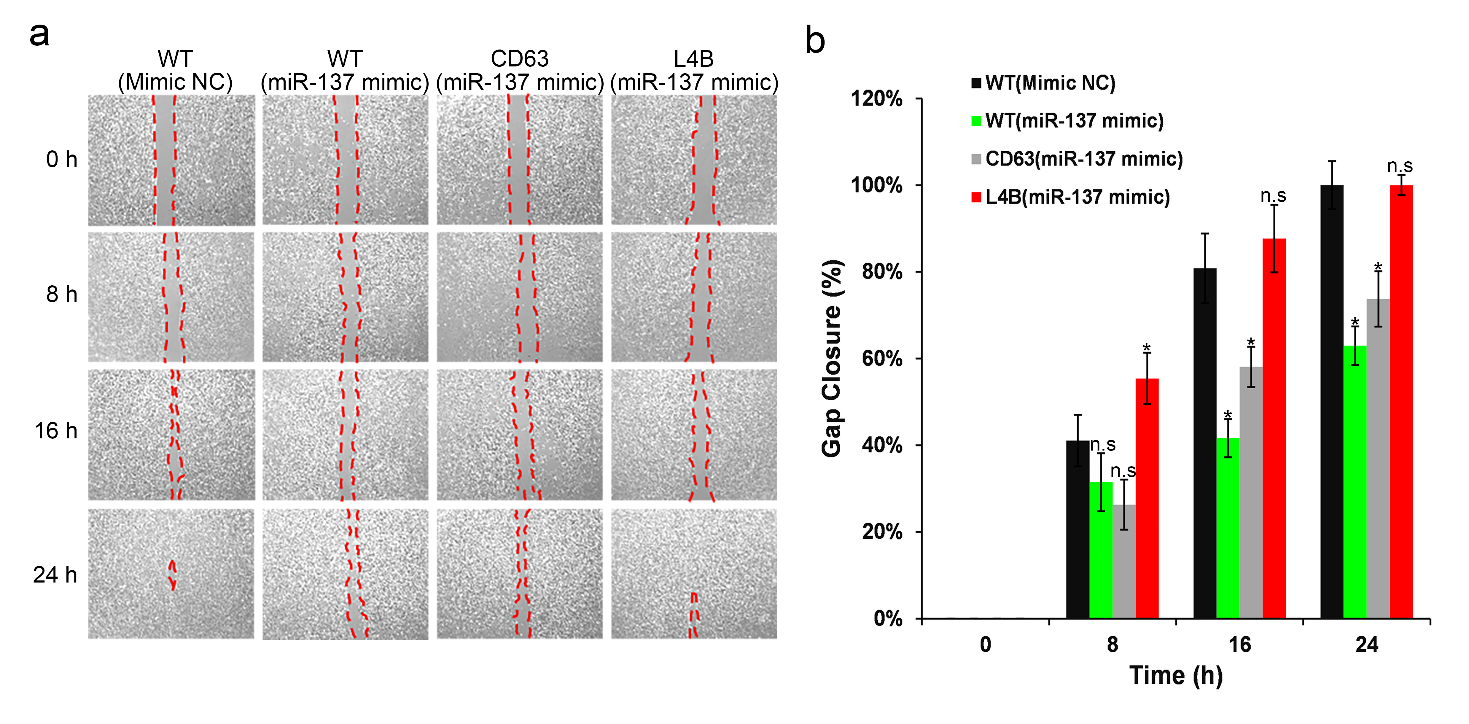
**

**Supplementary Figure S8. Wound-healing experiments indicates that miR-137 inhibits cell migration via targeting LAPTM4B**

1. WT, LAPTM4B- or CD63-stably expressing U2OS cells were transfected with miR-137 mimic or the Mimic NC, and cells were subjected to a wound-healing assay. Images are representative of five independent experiments at indicated time points. The red dashed line indicates the wound edge.
2. Quantification of wound closure of cells treated as in (a).

**
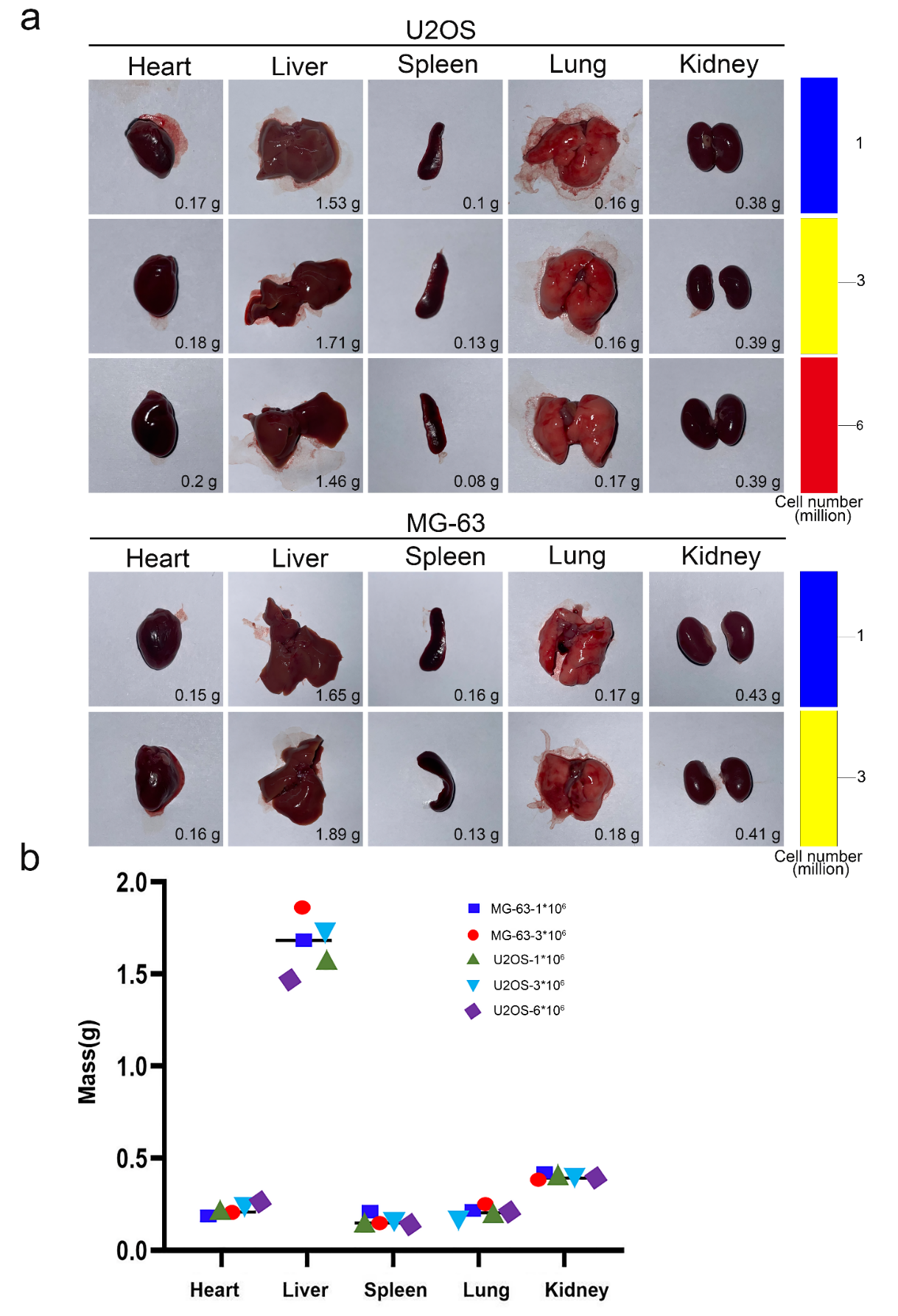
**

**Supplementary Figure S9. No metastatic nodule observed in U2OS and MG-63 injected nude mice**

1. A series range of 1*10^6^-6*10^6^ U2OS cells or MG-63 cells were injected into the tail vein of BABL/c female nude mice. The 6*10^6^ MG-63 injected mice did not survive afterward. The remaining mice were sacrificed six weeks after the injection, the organs were imaged for visualizing the metastasis nodules.
2. Mass of all the organs from the mice in the current study.

**Supplementary Table S1: All the primers used in this study**

| **mRNA expression analysis**  *LAPTM4B*  *RhoA*  *GAPDH* | **Forward primers (5'-3');**  **Reverse primers (5'-3')**  CCTGGATCATCCCATTCTTCTGT  AATTAGGAGGCAGTTGCCGTATG  TGCAAGCTAGACGTGGGAAG  AATTAGCGCCTGGTGTGTCA  GAAGGTGAAGGTCGGAGTC  GAAGATGGTGATGGGATTTC |
| --- | --- |
| **DNA copy number analysis**  *LAPTM4B-CN* | **Forward primers (5'-3');**  **Reverse primers (5'-3')**  AGCAAGCAAGAGAGTGGGAC  TTACTGCAAGCGGCTTACCT |

For miRNAs expression analysis, forward primers of miR-137, miR-128, miRNA reverse primer, as well as the internal control U6, were purchased from Guangzhou RiboBio Co., Ltd.
