# Supplementary material for: miR-137–LAPTM4B regulates cytoskeleton organization and cancer metastasis via the RhoA-LIMK-Cofilin pathway in osteosarcoma
Source: Oncogenesis. 2023 May 6;12(1):25. doi: 10.1038/s41389-023-00471-5 (PMC10163001; doi:10.1038/s41389-023-00471-5)
Supplement: Supplementary file 2 — Supplemental Materials and Methods [file 41389_2023_471_MOESM2_ESM.docx]

**miR-137–LAPTM4B regulates cytoskeleton organization and cancer metastasis via the RhoA-LIMK-Cofilin pathway in osteosarcoma**

Ruyu Yan^1,#^, Dan Liu^1,#^, Junjie Wang^1^, Minxia Liu^1,2^, Hongjuan Guo^1^, Jing Bai^1^, Shuo Yang^3^,Jun Chang^3^, Zhihong Yao^4^, Zuozhang Yang^4^, Tomas Blom^5,6*^, Kecheng Zhou^1,5,6*^

^1^ School of Life Sciences, Anhui Medical University, Hefei, 230032, China

^2^ Institute for Molecular Medicine Finland, Helsinki Institute of Life Science, University of Helsinki, Helsinki, 00290, Finland

^3^ Department of Orthopaedics, The First Affiliated Hospital of Anhui Medical University, Hefei, 230032, China

^4^ Bone and Soft Tissue Tumours Research Centre of Yunnan Province, Department of Orthopaedics, The Third Affiliated Hospital of Kunming Medical University (Yunnan Cancer Hospital, Yunnan Cancer Center), Kunming, Yunnan, 650118, China

^5^ Department of Anatomy, Faculty of Medicine, University of Helsinki, Helsinki, 00014, Finland

^6^ Minerva Foundation Institute for Medical Research, Helsinki, 00014, Finland

^#^ Ruyu Yan and Dan Liu contribue equally to the current study

*Corresponding author

Prof. Dr. Kecheng Zhou

Group Leader of “Cancer Metabolism Laboratory”

School of Life Science, Anhui Medical University

81 Meishan Road, 230032, Hefei, China

E-mail: zhoukecheng@ahmu.edu.cn

Dr. Tomas Blom

Department of Anatomy

Faculty of Medicine, University of Helsinki

Haartmaninkatu 8, 00014, Helsinki, Finland

E-mail: tomas.blom@helsinki.fi

**Supplemental Materials and Methods**

**Transient transfection of miRNA mimics or ASO**

MiRNA mimics, 2’-O-methylated miRNA antisense oligonucleotides (ASO), and their cognate controls were purchased from LabOmics. The transfection was performed using Hiperfect transfection reagent (QIAGEN, Cat#301705) following the manufacturer’s instructions. For the transfection, 20 nmol/L miRNA mimic and 40 nmol/L ASO were used.

**LAPTM4B 3’-UTR construction and luciferase reporter assay**

To obtain the luciferase construct, the full-length 3’-UTR of LAPTM4B was amplified from the genomic DNA inserted in the pEZX-MT01 (Gene Copoeia), which expresses Firefly luciferase and Renilla luciferase as a dual reporter gene. For the luciferase assays, miRNA mimic was co-transfected into the cells with the pEZX-MT01 plasmid containing 3’-UTR of LAPTM4B using Lipofectamine LTX with PLUS reagent (Invitrogen, Cat#15338100). The empty pEZX-MT01 plasmid was used as a negative control. After transfection, the luciferase activities were measured using Dual-Glo® Luciferase Assay System (Promega, Cat#2920) according to the manufacturer’s instructions.

**Western blotting**

To measure cellular protein, cells were washed with ice-cold PBS, lysed in RIPA lysis buffer (Beyotime, Cat#P0013B). To measure the phosphorylated protein, we used SDS boiling buffer (2.5% SDS, 250 mM Tris/HCl pH 6.8, including 50 mM NaF, 10 mM b-glycerophosphate, 0.5 mM DTT, 0.5 mM PMSF), as previously described^1^.

After cells lysed on ice for 30 min, we boiled the cell lysates at 99^o^C for 10 min. Equal amounts of proteins were resolved on 12% Mini-Protean TGX Stain-Free gels (Bio-Rad, Cat#161-0185) and transferred onto LF-PVDF (Bio-Rad, Cat#170-4274) or NC Transfer Membrane (MERCK, Cat# HATF00010). Membranes were blocked with 3% BSA in TBS containing 0.1% Tween-20 (TBST) for 1 h at room temperature or 5 % non-fat milk in TBST, and subsequently probed with primary antibodies at 4^o^C overnight. After washing four times with TBST, membranes were incubated with secondary antibodies for 1 h at room temperature. Membranes were washed, incubated with ECL Clarity (Bio-Rad, Cat#170-5060), and imaged with a ChemiDoc^TM^ MP Imaging System (Bio-Rad, Cat#17001402). The quantifications were done either by normalizing to total protein content using Image Lab software (version 5.2.1; Bio-Rad) and the Stain-Free technology, or by normalizing to internal control protein using ImageJ software version 1.53C (NIH, Bethesda, MD; http://imagej.nih.gov/ij).

**Analysis of RhoA ubiquitination**

Cells were grown on 60-mm dishes, and were transfected with LAPTM4B siRNA or Ctrl siRNA with 20 nM as the final concentration. 72 h after transfection, cells were scraped in RIPA lysis buffer (Beyotime, Cat#P0013D) containing protease inhibitor cocktail (MCE, Cat#HY-K0010), cells were treated with 20 µM MG-132 for 9 h before the harvest. The lysates were cleared by centrifugation at 12,000 x g for 10 min at 4 ^o^C. Equal amounts of cleared cell lysates were incubated with 2 µg of primary RhoA antibody (Santa-Cruz, Cat#sc-418) for 3 h at 4 ^o^C on a rocker platform. 30 µl resuspended protein A/G agarose beads (Santa Cruz, Cat#sc-2003) were then added and incubated at 4 ^o^C overnight. Afterwards, immunoprecipitates were enriched by centrifugation and wash 2 X 10 min with 1 ml RIPA. The supernatant was aspirated and discarded, the pellet was resuspended in 40 µl 1X Laemmli Sample Buffer and afterwards boiled at 99^o^C for 10 min. The precipitated proteins were analyzed by western blotting for measuring RhoA ubiquitination.

**Hematoxylin and Eosin (H&E) staining and Immunohistochemistry (IHC) staining**

Paraffin-embedded tissue samples were sectioned (3 µm) for further experiments. HE staining was automatically conducted by Autostainer (Leica, Cat#CV5030), with hematoxylin for staining cell nuclei and eosin for cytoplasm.

IHC experiments were done as the following description. Tissue sections were oven-dried and dewaxed with xylene, rehydrated with gradient anhydrous ethanol, and repaired by boiling with sodium citrate antigen repair solution for 2 min, followed by rinsing with H_2_O to room temperature. The sections were blocked with 0.3% H_2_O_2_ for 10 min and incubated with anti-LAPTM4B monoclonal antibody (Atlas Antibodies, Cat#AMAb91356,1:600) for 2.5 h at room temperature. We afterward washed sections with PBS and incubated them with a secondary HRP-conjugated antibody (MXB, Cat#KIT-5010) for 30 min at room temperature. Finally, the sections were stained for 2 min using the DAB development kit (Proteintech, Cat#PR30010) and hematoxylin for 10 sec. The slices were dehydrated with gradient anhydrous ethanol and then sealed with neutral resin gel.

For the IHC analysis, ten random fields of vision per sample were taken by microscopy (Leica, Cat#DM2500), and were further quantified via “IHC Profiler” in ImageJ software version 1.53C (NIH, Bethesda, MD; http://imagej.nih.gov/ij). Weak immunoreactivity of osteoblasts was detected. The low cellularity in healthy bone tissues was also considered and the IHC signal was quantified via normalizing to the total cell number per image. All the H&E and IHC images were assessed by two independent pathologists.

**Immunofluorescence staining and confocal microscopy**

Immunofluorescence staining was performed as described previously^1,2^. Briefly, we fixed cells in coverslips with 4% paraformaldehyde in PBS at room temperature for 20 min, then quenched cells in 50 mM NH_4_Cl at room temperature for 10 min. We further washed coverslips with PBS and permeabilized cells with 0.1% Triton X-100 in PBS for 10 min, blocked in 10% FBS in PBS for 30 min, and then incubated with anti-LAPTM4B (Atlas Antibodies, Cat#AMAb91356, 1:300) at 37 ^o^C for 45 min. Cells were further washed in PBS and incubated with secondary antibody (ThermoFisher Scientific, Cat#A-11001, 1:200) for 45 min at 37^o^C. Finally, we washed coverslips in PBS, rinsed in MQ-H_2_O, and mounted on microscope slides using Mowiol/ DABCO (Calbiochem, Cat#475904/ Sigma, Cat#D-2522). Images were captured with Leica TCS SP8X confocal microscope and analyzed using ImageJ. For visualization of F-actin, Alexa Fluor 568 Phalloidin (Molecular probes, Cat#A-12380, dilution 1:50) was added during the secondary antibody incubation step.

**Analysis of cell area and transverse arc numbers**

The images captured by confocal microscopy were further used for quantifying spread area and transverse arc numbers by using modified protocols described previously^3,4^. Briefly, we first converted cell images into greyscale, conducted top-hat transformation by illumination correction, and next defined background via global threshold. These images were further processed as follows: gauss filter, path opening, directional top-hat, hysteresis threshold, and processed images were next traced to find stress fiber connections. The produced information was then used to calculate and evaluate stress fiber number and pattern.

**Cell migration analysis**

Cell migration was measured by the xCELLigene system^5^ and the wound-healing assay.

Real-time and label-free cell migraiton measurement by xCELLigene system was adopted from our previous study^7^. Wild-type, LAPTM4B stably expressing, and CD63 stably expressing U2OS cells were transfected with miRNA mimics, on the following day after transfection, cells were trypsinized, and 6×10^4^ cells were seeded in the top chamber of CIM-plate 16 (ACEA, Cat#5665817007) with serum free medium, complete medium containing 10% FBS in the down chamber was served as cell migration chemoattractant. CIM plate equilibrium was done at 37 ^o^C incubator for 60 min, the plate background were measured after equilibrium. After seeding the cells, we keep CIM plate at room temperature for 30 min to settle down cells on the bottom surface of the top chamber. Afterwards, we start measuing cell migration every 15 min for consecutive 25 h.

The wound-healing assay was performed as described previously^6^. Briefly, wild-type, LAPTM4B stably expressing, and CD63 stably expressing U2OS cells were transfected with miRNA mimics, then grown in Ibidi culture insert chambers (Ibidi, Cat#80209) for 3 days. The insert was gently removed, and the medium was then supplemented with 10 mg/ml mitomycin C (Cell signaling technology, Cat#51854). Wound closure was imaged by phase-contrast microscopy (Olympus, Cat#CKX41) at the indicated time points (from 0 to 24 h).

**Reference**

1 Zhou K, Dichlberger A, Martinez-Seara H, Nyholm TKM, Li S, Kim YK *et al.* A Ceramide-Regulated Element in the Late Endosomal Protein LAPTM4B Controls Amino Acid Transporter Interaction. *ACS Cent Sci* 2018; 4: 548–558.

2 Blom T, Li S, Dichlberger A, Bäck N, Kim YAYA, Loizides-Mangold U *et al.* LAPTM4B facilitates late endosomal ceramide export to control cell death pathways. *Nat Chem Biol* 2015; 11: 799–806.

3 Rogge H, Artelt N, Endlich N, Endlich K. Automated segmentation and quantification of actin stress fibres undergoing experimentally induced changes. *J Microsc* 2017; 268: 129–140.

4 Elosegui-Artola A, Jorge-Peñas A, Moreno-Arotzena O, Oregi A, Lasa M, Garcéa-Aznar JM *et al.* Image Analysis for the Quantitative Comparison of Stress Fibers and Focal Adhesions. *PLoS One* 2014; 9: e107393.

5 Bird C, Kirstein S. Real-time, label-free monitoring of cellular invasion and migration with the xCELLigence system. *Nat Methods* 2009; 6: 1–2.

6 Liu M, Yan R, Wang J, Yao Z, Fan X, Zhou K. LAPTM4B-35 promotes cancer cell migration via stimulating integrin beta1 recycling and focal adhesion dynamics. *Cancer Sci* 2022; 113: 2022–2033.

7 Zhou K, Dichlberger A, Ikonen E, Blom T. Lysosome Associated Protein Transmembrane 4B (LAPTM4B)-24 is the predominant protein isoform in human tissues and undergoes rapid, nutrient-regulated turnover. *Am J Pathol* 2020; 190: 2018–2028.
